# Crystal Polymorph Search in the *NPT* Ensemble via a Deposition/Sublimation Alchemical Path

**DOI:** 10.1021/acs.cgd.3c01358

**Published:** 2024-03-09

**Authors:** Aaron J. Nessler, Okimasa Okada, Yuya Kinoshita, Koki Nishimura, Hiroomi Nagata, Kaori Fukuzawa, Etsuo Yonemochi, Michael J. Schnieders

**Affiliations:** † Department of Biomedical Engineering, University of Iowa, 103 South Capitol Street, 5601 Seamans Center for the Engineering Arts and Sciences, Iowa City, Iowa 52242, United States; ‡ Sohyaku Innovative Research Division, Mitsubishi Tanabe Pharma Corporation, 1000 Kamoshida-cho, Aoba-ku, Yokohama, Kanagawa 227-0033, Japan; § Analytical Development, Pharmaceutical Sciences, Takeda Pharmaceutical Company Limited, 2-26-1, Muraoka-Higashi, Fujisawa 251-8555, Kanagawa, Japan; ∥ CMC Modality Technology Laboratories, Production Technology and Supply Chain Management Division, Mitsubishi Tanabe Pharma Corporation, Osaka 541-8505, Japan; ⊥ Graduate School of Pharmaceutical Sciences, Osaka University, 1-6 Yamadaoka, Suita, Osaka 565-0871, Japan; # Department of Physical Chemistry, School of Pharmacy and Pharmaceutical Sciences, Hoshi University, 2-4-41 Ebara, Shinagawa-ku, Tokyo 142-8501, Japan; ¶ Department of Biochemistry, University of Iowa, 51 Newton Road, 4-403 Bowen Science Building, Iowa City, Iowa 52242, United States

## Abstract

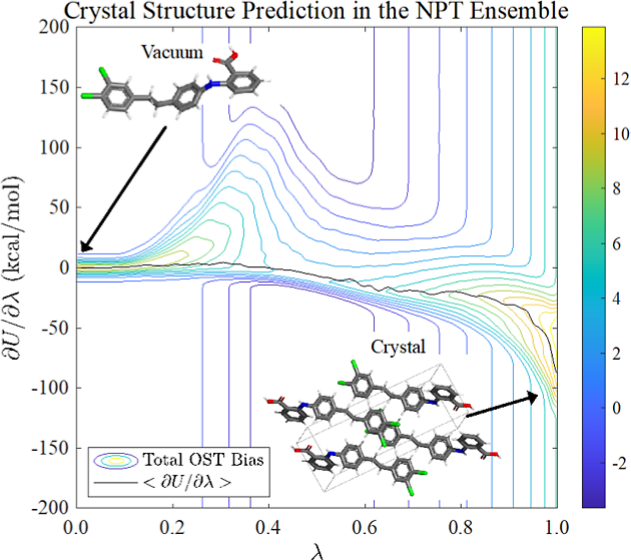

The formulation of active pharmaceutical ingredients involves discovering stable crystal packing arrangements or polymorphs, each of which has distinct pharmaceutically relevant properties. Traditional experimental screening techniques utilizing various conditions are commonly supplemented with in silico crystal structure prediction (CSP) to inform the crystallization process and mitigate risk. Predictions are often based on advanced classical force fields or quantum mechanical calculations that model the crystal potential energy landscape but do not fully incorporate temperature, pressure, or solution conditions during the search procedure. This study proposes an innovative alchemical path that utilizes an advanced polarizable atomic multipole force field to predict crystal structures based on direct sampling of the *NPT* ensemble. The use of alchemical (i.e., nonphysical) intermediates, a novel Monte Carlo barostat, and an orthogonal space tempering bias combine to enhance the sampling efficiency of the deposition/sublimation phase transition. The proposed algorithm was applied to 2-((4-(2-(3,4-dichlorophenyl)ethyl)phenyl)amino)benzoic acid (Cambridge Crystallography Database Centre ID: XAFPAY) as a case study to showcase the algorithm. Each experimentally determined polymorph with one molecule in the asymmetric unit was successfully reproduced via approximately 1000 short 1 ns simulations per space group where each simulation was initiated from random rigid body coordinates and unit cell parameters. Utilizing two threads of a recent Intel CPU (a Xeon Gold 6330 CPU at 2.00 GHz), 1 ns of sampling using the polarizable AMOEBA force field can be acquired in 4 h (equating to more than 300 ns/day using all 112 threads/56 cores of a dual CPU node) within the Force Field X software (https://ffx.biochem.uiowa.edu). These results demonstrate a step forward in the rigorous use of the *NPT* ensemble during the CSP search process and open the door to future algorithms that incorporate solution conditions using continuum solvation methods.

## Abstract 2

Formulation of active pharmaceutical ingredients is generally accomplished by experimental screening techniques utilizing various conditions. These screenings are commonly supplemented with in silico crystal structure prediction to inform the crystallization process and mitigate risk. Predictions are often based on advanced classical force fields or quantum mechanical calculations that model the crystal potential energy landscape but do not fully incorporate temperature, pressure, or solution conditions during the search procedure. This study proposes an innovative alchemical path that utilizes an advanced polarizable atomic multipole force field to predict crystal structures based on direct sampling of the *NPT* ensemble.

## 1 Introduction

The successful translation of an active pharmaceutical ingredient (API) into a viable therapeutic is supported by its formulation as a bioavailable solid form. However, many APIs can exist in multiple stable crystal arrangements, known as polymorphs, with significantly different pharmaceutically relevant properties such as thermodynamic stability, solubility, dissolution rate, and processability.^1−5^ Polymorphs can be discovered via screening under a variety of experimental conditions (e.g., solvent, pH, salt, temperature, and pressure). Despite significant recent progress in computational crystal structure prediction (CSP) as evidenced by successful blind tests, accurate polymorph prediction for complex molecules remains challenging.^6−14^ This is particularly true for large, flexible APIs and those with complex asymmetric units that contain coformers and/or water molecules.^15^

Traditional CSP protocols involve an exhaustive search over various configurations, from space groups and molecule arrangements to internal conformations and unit cell parameters. Efficient force fields and density functional theory (DFT) calculations^16^ are often employed to guide this search, but incorporating thermal effects has typically been limited to a small subset of final candidates.^17−21^ Here, we present a groundbreaking approach for CSP that directly samples from the thermodynamically relevant *NPT* ensemble, allowing us to efficiently generate all potential polymorphs, including those stabilized by the temperature and pressure. This paves the way for efficient prediction of polymorphs using the crystal free energy landscape instead of the potential energy landscape, with implications for both the formulation of pharmaceuticals and materials science.

Typically, bespoke or tailor-made force fields are created for the CSP search^22^ that go beyond the accuracy of fixed partial charge models such as the general Amber force field (GAFF),^23^ CHARMM general force field (CGenFF),^24^ Open Force Field (OpenFF),^25^ or OPLS-AA (via LigParGen).^26^ The advantages of replacing charges with fixed atomic multipoles were observed in the first blind test in 1999,^27^ although it was recognized early on that the improved accuracy observed for relatively rigid molecules can be lost in the context of flexible molecules due to lack of conformational transferability of the electrostatic moments.^28,29^ One approach to mitigate this challenge is to calculate a unique distributed multipole analysis^30−32^ (DMA) for each conformation, followed by constraint of the crystal optimization to use rigid body coordinates as implemented originally in DMAFlex.^33^ More recently, rigid body optimization using a pairwise potential (limited to fixed partial charges) fit to symmetry-adapted perturbation theory (SAPT) has shown promise for predicting the crystal structures of relatively rigid molecules.^12^

Alternatively, changes in multipole moments as a function of conformation can be described by incorporating an explicit many-body polarization response, which has been demonstrated by the polarizable Atomic Multipole Optimized Energetics for Biomolecular Applications (AMOEBA) force field in the context of X-ray crystallography refinement^34−38^ and the prediction of the structure, thermodynamic stability, and solubility of organic crystals.^39−41^ While the AMOEBA model offers intramolecular flexibility to support molecular dynamics and the calculation of free energy differences based on a consistent treatment of intra- and intermolecular polarization,^42^ the DMACRYS software implements a complementary approach that includes anisotropic polarizability and permanent multipoles based on constraining to rigid molecule degrees of freedom.^43^ Notable progress using permanent multipoles in the context of intramolecular flexibility has also been demonstrated within the GRACE software, although the current model lacks explicit treatment of electronic polarization.^13^ This work takes a step forward by leveraging the full potential of AMOEBA within a novel *NPT* ensemble approach that directly connects the vacuum and crystalline phases through an alchemical path. This not only enables efficient polymorph prediction under varied temperature and pressure conditions but also facilitates the exploration of the accuracy and transferability of the automated AMOEBA parametrization framework.^44^

While alchemical free energy simulations are helping to revolutionize drug discovery,^45,46^ their application in CSP has been limited. This is partly due to the lack of efficient connections between the vacuum and crystalline states. We describe a novel alchemical path that bridges this gap by using nonphysical intermediates that optimize the efficiency of the polymorph search. This thermodynamic path exploits the state function property of free energy by traversing carefully designed intermediate states that simplify the search landscape. Specifically, our approach within Force Field X (FFX)^47^ focuses on the crystal’s asymmetric unit instead of the entire unit cell.^38^ Simulation of the phase transition using a single asymmetric unit dramatically accelerates calculation of the energy and forces while simultaneously reducing the phase space volume (as summarized in Table 1 for compound XXIII). To enhance sampling of the deposition (or sublimation) phase transition,^39^ we leverage the orthogonal space tempering (OST) method,^48^ flattening the free energy surface for smoother exploration.^49,50^ Additionally, a novel Monte Carlo barostat^51^ ensures that fluctuations of unit cell parameters are consistent with the *NPT* ensemble while respecting lattice constraints. Each component of this tailored path is detailed in the Methods section.

**Table 1:** Comparison of Asymmetric Unit (ASU), Unit Cell (UC), and Replicated Unit Cell Simulations for Compound XXIII^a^

space group	number of molecules	degrees of freedom	time for energy and forces (s)
*P*1̅			
ASU	1	129	0.012
*P*1 UC	2	258	0.017
*P*1 3 × 3 × 3 UC	54	6966	0.303
*P*2_1_/*c*			
ASU	1	129	0.013
*P*1 UC	4	516	0.030
*P*1 3 × 3 × 2 UC	72	9288	0.431

a The efficiency of sampling the ASU phase transitions is due to both the reduction of phase space volume and fast evaluation of the energy and forces. The replicate crystal dimensions for both cases (e.g., 3 × 3 × 3 for *P*1) are the smallest values consistent with the use of the minimum image convention.

Once candidate polymorphs have been collected, the goal of CSP pipelines is generally to eliminate duplicates using crystal packing comparisons^52^ and/or clustering, followed by rescoring a subset of the most favorable polymorphs (e.g., using DFT).^53^ As the discrepancies between the potential energy function used during the search phase (e.g., the AMOEBA force field) and the electronic structure method are reduced, fewer polymorphs need to be rescored. Although our team has recently demonstrated the alchemical *NPT* approach for a series of six molecules,^54^ here we focus on describing our novel pipeline in detail for 2-((4-(2-(3,4-dichlorophenyl)ethyl)phenyl)amino)benzoic acid (XAFPAY),^55^ which is known as compound XXIII from the 2015 blind CSP test.^11^ Compound XXIII is a flexible molecule with three rotatable bonds that proved challenging as only two teams successfully predicted all the three *Z*′ = 1 polymorphs. Furthermore, it was also chosen because it exhibits high druglikeness^56^ and facilitates comparisons to other notable CSP approaches. We delve deeper into the specifics of our pipeline and its application to compound XXIII in the following sections.

## 2 Methods

### 2.1 Polarizable AMOEBA Force Field

The simulations in this work leverage the AMOEBA^57^ force field for organic molecules, which combines bonded and nonbonded interactions.
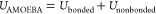
1

The bonded terms include bond stretching, angle bending, bond–angle cross-terms, out-of-plane bending, and torsions.
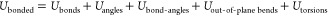
2

The nonbonded terms include van der Waals interactions, permanent atomic multipoles truncated at quadrupole order, and induced dipoles.
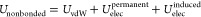
3

This functional form is implemented within the open source FFX software^47^ (https://ffx.biochem.uiowa.edu) in a manner that supports space group symmetry operators^38^ in the context of smooth particle-mesh Ewald (PME) summation^58^ for long-range multipolar electrostatics.^59^ Buffered 14–7 van der Waals interactions^60^ were smoothly truncated at 12 Å, including softcore support compatible with the OST method.^39^ PME used an Ewald coefficient of 0.545, a real space cutoff of 7 Å, a B-spline order of 5, and a reciprocal space mesh density of at least 1.2 grid points per Angstrom along each axis (for small unit cells, a minimum of 16 grid points along each axis was enforced). The induced dipole self-consistent field was solved using a preconditioned conjugate gradient solver^61^ to achieve the convergence criteria of 1.0 × 10^–6^ rms debye (i.e., convergence is reached once the rms change in induced dipoles between cycles is less than 1.0 × 10^–6^ debye).

### 2.2 Force Field Parameters and Initial Coordinates

Three-dimensional molecular coordinates of compound XXIII were obtained from the PubChem^62^ database in SDF format. AMOEBA force field parameters were produced using both the initial release of the Poltype parametrization tool^63^ and a recently described second version called Poltype2.^44^ Poltype2 leverages either Gaussian^64^ or PSI4^65^ for quantum mechanical (QM) calculations and Tinker^66^ for a variety of molecular mechanics (MM) calculations. Following local QM optimization at the MP2/6-31G* level of theory (with dihedrals of rotatable bonds restrained to an extended conformation), Stone’s Gaussian distributed multipole analysis (GDMA)^32^ is used to assign initial atomic multipole moments through quadrupole order using a QM electron density calculated at the MP2/6-311G** level of theory. The Tinker^66^*Potential* program is used to further optimize the permanent multipole moments from GDMA to match the QM electrostatic potential (at the MP2/aug-cc-pvtz level of theory) outside of the molecule. Valence parameters and atomic polarizabilities are obtained from a Poltype2 database. Poltype torsional parameters that were missing from its database were fit against QM energies as described previously,^44^ whereas for Poltype2, all torsional parameters were fit. Atomic coordinates from Poltype local optimization were used to prepare for the CSP search via the FFX command *PrepareSpaceGroups*, which places coordinate and property files into subdirectories for each space group populated above a specified probability cutoff (0.5% for this work) in the Cambridge Structural Database (CSD).^67^

The AMOEBA parameters used for the alchemical polymorph search described below were generated using the first version of Poltype in the summer of 2020 (abbreviated as AP20 for AMOEBA Poltype 2020). The permanent atomic multipole parameters generated for organochlorine atoms were improved following a systematic study of σ-hole effects^68^ and made available in Poltype2. Rerunning Compound XXIII with Poltype2 in the summer of 2023 (abbreviated as AP23) resulted in chloride multipole moments with negative monopoles that concord more closely with published values^68^ as described in Table 2. The updated AP23 parameters used the newer grid-based GDMA algorithm with nonbonded parameters adjusted to reproduce hydration free energies and all torsions for each rotatable bond fit to QM. Notably, the monopole AP20 parameter was too positive, which results in overly favorable interactions with carboxyl groups within some packing arrangements. The AP23 parameters show improved concordance to DFT lattice energies, as described in the Results section. Both the AP20 and AP23 parameter files are supplied as the Supporting Information.

**Table 2:** Compound XXIII AMOEBA Permanent Atomic Multipole Moments and Polarizability for Organochlorine Atoms from Poltype (AP20) and Poltype2 (AP23)^a^

source	monopole	dipole	quadrupole			polarizability (Å^3^)
Compound XXIII						
AP20 *ortho* Cl	0.04870	–0.02736	–0.77592	0.00000	–0.02542	2.5000
		0.00000		–0.87950	0.00000	
		0.33726			1.65542	
AP20 *para* Cl	0.04990	0.02518	–0.74656	0.00000	0.02356	2.5000
		0.00000		–0.90390	0.00000	
		0.34207			1.65046	
AP23 *ortho* Cl	–0.39652	0.13928	–0.79877	0.00000	0.24618	2.5670
		0.00000		–0.37716	0.00000	
		–0.25266			1.17593	
AP23 *para* Cl	–0.39484	0.13917	–0.79616	0.00000	0.24905	2.5670
		0.00000		–0.36705	0.00000	
		–0.26462			1.16321	
CH_3_Cl^68^						
Cl	–0.22443	0.00000	–0.88958	0.00000	0.00000	2.5000
		0.00000		–0.88997	0.00000	
		0.17165			1.77955	

a Literature values for the chlorine permanent atomic multipole moments for CH_3_Cl^68^ and the polarizability of aromatic chlorine are included for comparison. The multipole moments are each in their respective atomic units.

### 2.3 Alchemical Path Connecting Vacuum and Crystalline States

**Figure 1:**
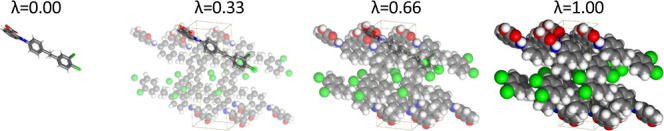
Smooth alchemical path connects vacuum (λ = 0) and crystalline (λ = 1) states. During simulations in the *NPT* ensemble, a random walk along state variable λ facilitates sampling of both atomic coordinates and lattice parameters. Here, the path sampled for compound XXIII in the *P*1 space group is depicted.

Generalized ensemble (GE) free energy simulation methods can be employed to facilitate the crossing of free energy barriers. Here, GE simulations are utilized to sample the free energy surface along state parameter λ that connects vacuum and crystalline phases via an alchemical path.^69^ At λ = 0, the molecule(s) (i.e., all components of the asymmetric unit) is fully uncoupled from surrounding symmetry mates to achieve the vacuum state. As the state parameter increases during the GE simulation, symmetry mate interactions are smoothly restored until the molecule(s) are fully interacting with the crystalline environment at λ = 1 as described previously for *NVT* simulations^39^ and depicted in Figure 1 for *NPT* simulations.

### 2.4 Orthogonal Space Tempering

Although a first-order GE method eliminates barriers along the λ path between vacuum and crystalline states (e.g., metadynamics^70^), “hidden” free energy barriers can exist perpendicular to this path that hinder exhaustive exploration of the crystalline free energy surface.^71^ One common example of a barrier that is not aligned with the alchemical λ path between phases is rotation about a torsion. The traversal of torsional barriers becomes particularly important when considering larger, more flexible APIs as unique polymorphs may have drastically different configurations, which necessitates the crossing of all hidden barriers for an exhaustive search of the free energy landscape. To ameliorate such hidden barriers and ultimately cross them, this work employs a second-order GE simulation method called orthogonal space tempering^48^ that combines transition-tempered metadynamics^72^ with the orthogonal space random walk method.^49^ The total potential energy *U*_OST_(λ,**x**) is the sum of the AMOEBA force field and a time-dependent bias to yield
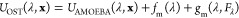
4where the *g*_m_(λ,*F*_λ_) bias is a sum of two-dimensional repulsive potentials given by
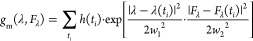
5that is used to define a 1D bias *f*_m_(λ) via thermodynamic integration
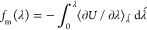
6of the quantity
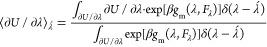
7with β = 1/(*k*_B_*T*). Tempering defines a nonconstant bias height *h*(*t*_*i*_) that decays as the simulation progresses. As the bias progressively flattens the path, *h*(*t*_*i*_) decreases asymptotically to 0 based on the expression
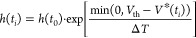
8

The tempering threshold, *V*_th_, requires the entire path be at least lightly covered with bias before the transition to tempering begins (*V*_th_ = 1 kcal/mol in this work). If *V*_th_ = 0, tempering begins immediately (i.e., no threshold must be reached). Exponential decay is controlled by parameter Δ*T*, which was set to 2 k_B_T here, and the expression
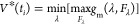
9where the max operation, for each fixed λ, is over the range of *F*_λ_ values (i.e., the 2D *g*_m_ histogram is reduced to a 1D function of λ), followed by the min operation over λ. In this work, the two-dimensional bias histogram was defined by lambda bins of width 0.005 and *F*_λ_ bins of width 2 kcal/mol. The initial Gaussian bias height *h*(*t*_0_) can be set to ∼0.02–0.05 kcal/mol (an initial bias height of 0.05 kcal/mol was used in this work), with standard deviations equal to two bins in either dimension (*w*_i_ = 0.01 and *w*_2_ = 4.0 kcal/mol) and truncated after five bins during evaluation of the 2D bias.

### 2.5 Monte Carlo Barostat Samples Lattice Parameters at Constant Pressure

To sample polymorphs from the isothermal–isobaric (*NPT*) ensemble, atomic coordinates are propagated, and temperature is maintained using stochastic dynamics,^69,73^ while lattice parameters fluctuate based on a customized Monte Carlo (MC) barostat^51^ that respects the lattice system constraints given in Table 3. The MC trial moves depend on a simple set of parameters that include.

(1) Minimum (0.75 g/cm^3^) and maximum (1.6 g/cm^3^) density constraints.
(2) Maximum volume (*v*_max_ = 1 Å^3^) and angle (*a*_max_ = 0.5°) moves.
(3) Trial move frequency (every 10 molecular dynamics steps on average).

**Table 3:** Lattice System Constraints Maintained by the Monte Carlo Barostat

lattice system	constraints		degrees of freedom
	lengths	angles	
triclinic	none	none	6
monoclinic	none	*a* = γ = 90°	5
orthorhombic	none	α = β = γ = 90°	4
tetragonal	*a* = *b*	α = β = γ = 90°	2
rhombohedral	*a* = *b = c*	α = β = γ	2
hexagonal	*a* = *b*	α = β = 90°, γ = 120°	2
cubic	*a* = *b* = *c*	α = β = γ = 90°	1

The density limits avoid inefficient sampling of unphysical lattice parameters as the simulation approaches and enters the vacuum state (λ = 0) where density changes do not alter the potential energy. The maximum volume and angle movements can be tuned to achieve a target acceptance rate. Finally, the trial move frequency serves to maintain efficiency by only adding a single extra energy evaluation every ∼10 molecular dynamics steps, such that pressure control slows the simulation by only ∼10%.

To generate a proposed change to one or more lattice parameters, first a single degree of freedom is chosen at random (e.g., for a triclinic cell, there are six possibilities, while for an orthorhombic cell, there are only four possibilities). If a lattice axis is chosen, then the proposed change is based on a pseudorandom number, *x*, that is greater than or equal to 0.0 and less than 1.0, which is drawn from a (approximately) uniform distribution. The proposed change in the volume of the asymmetric unit is then calculated as Δ*V* = *V*_max_ (2*x* – 1). This volume change is achieved by scaling the chosen lattice length degree of freedom. For example, in the triclinic lattice system, either the *a*-, *b*-, or *c*-axis length is scaled in isolation. On the other hand, for the cubic lattice system, the *a*-, *b*-, and *c*-axis lengths are constrained to be equivalent and are scaled in tandem. If a lattice angle is chosen, then the proposed change (e.g., Δα) is also based on a pseudorandom number, *x*, to give Δα = *a*_max_ (2*x* – 1). After application of the proposed angle change to the lattice angle(s) in a manner that respects the lattice system constraints, the volume remains unchanged via uniform scaling of all lattice lengths.

The energy change of the asymmetric unit due to the proposed trial move is calculated as
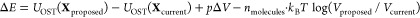
10where *p* is the target pressure, *n*_molecules_ is the number of molecules in the asymmetric unit, *k*_B_ is Boltzmann’s constant, and *T* is the absolute temperature. Atomic coordinates for the proposed lattice parameters (**X**_proposed_) are generated from the current atomic coordinates (**X**_current_) by keeping the fractional coordinates of the centers of mass for all molecules unchanged. The MC barostat move is accepted if Δ*E* < 0; otherwise, the probability of acceptance is given by e^(−Δ*E*/*k*_B_*T*)^.

### 2.6 Simulations and Resources

Each batch of simulations, using the FFX command *Thermodynamics*, was replicated five times and consisted of 100 jobs with 12 walkers each (1200 simulations) for the top 15 highest probability space groups (which accounts for all space groups that populate >0.5% of the deposited experimental structures) in the CSD (i.e., *P*1, *P*1, *P*2_1_, *C*2, *Cc*, *P*2/*c*, *P*2_1_/*c*, *C*2/*c*, *P*2_1_2_1_2_1_, *Pca*2_1_, *Pna*2_1_, *Pbcn*, *Pbca*, *Pnma*, and *H*3). Although a more complete analysis for this search might employ the top 43 space groups, the first 15 are sufficient to demonstrate the algorithm. As this is a retrospective analysis, the data presented here emphasize the space groups that have an experimentally determined polymorph: *P*1 and *P*2_1_/**c**. Direct polarization, in which sites are polarized by the field of the permanent multipoles only (i.e., the field due to induced dipoles does not contribute), was utilized to accelerate sampling, although the analysis presented in the results demonstrates that either no polarization or full convergence of the self-consistent field (SCF) offers superior concordance to DFT results. Simulations were limited to include a single molecule (*Z*′ = 1) in the asymmetric unit. A snapshot was saved every 10 ps conditional on λ being greater than 0.8 and the potential energy achieved by local minimization (to rms gradient of 0.1 kcal/mol/Å) being within 10 kcal/mol of the lowest energy structure previously observed. All simulations were performed on the Fugaku supercomputer at the Riken Center for Computational Science in Kobe, Japan. Each job was assigned one 48 (+2 assistant) core Armv8.2-A node with 32 GB of RAM. Using a single Fugaku CPU for approximately 9 h, 12 ns of sampling for compound XXIII was obtained based on executing 12 independent walkers that each sampled for 1 ns (each walker was allocated four CPU cores).

### 2.7 Filtering of Polymorphs Using Potential Energy and Density

The coordinates and lattice parameters of each *NPT* snapshot were minimized (using the FFX command *MinimizeCrystals*) under the AP20 force field to an rms gradient convergence criterion of 0.03 kcal/mol/Å. Predictions with sufficiently high density (>1.25 g/cm^3^) and favorable potential energy (within 10 kcal/mol of the most stable observed structure) were prioritized and kept for further analysis.

### 2.8 PAC Packing Metric

The PAC algorithm, which compares crystal structures using an efficient algorithm that prioritizes low radius of gyration molecular clusters, was used to compare all snapshots for a given space group.^52^ PAC (via the FFX command *SuperposeCrystals*) calculates the root-mean-square deviation (rmsd) between the heavy atoms of two crystals by optimally superimposing a cluster of *N* molecules (where *N* = 20 is denoted by rmsd_20_). PAC was used to compute a similarity matrix for all snapshots of each space group against themselves (e.g., all *P*1 snapshots vs all *P*1snapshots), which then served as input to the clustering algorithm described below. The comparisons also serve as a test for convergence because polymorphs that are encountered multiple times during the search are enumerated.

### 2.9 Iterative Hierarchical Clustering on the PAC rmsd_20_ Similarity Matrix

The clustering method (available via the FFX command *Cluster*) adopted by this pipeline is an iterative procedure in which crystals are clustered based on one-to-one similarity computed by PAC. The clustering method proceeds as follows:

(1) Randomly select a seed structure that has not been clustered.
(2) All structures that are not included in a cluster and are within an rmsd_*N*_ criteria (e.g., rmsd_20_ of 0.5 Å for this work) to the selected structure are classified in its cluster.
(3) Repeat steps 1 and 2 until all structures are clustered.

Repeat steps 1–3 multiple times (e.g., 1000) and adopt the clustering that results in a minimum number of clusters.

In this way, similar structures are excluded from further analysis, which includes expensive DFT calculations. The clustering does not incorporate energy values and is based solely on rmsd_20_. The 0.5 Å cutoff was chosen to error on the side of promoting structures relatively similar to DFT analysis. This method was compared to a nonhierarchical *K*-means clustering algorithm. While *K*-means clustering reduced the number of structures by approximately 40%, this iterative hierarchical method reduced the number of structures by around 90%.

**Figure 2:**
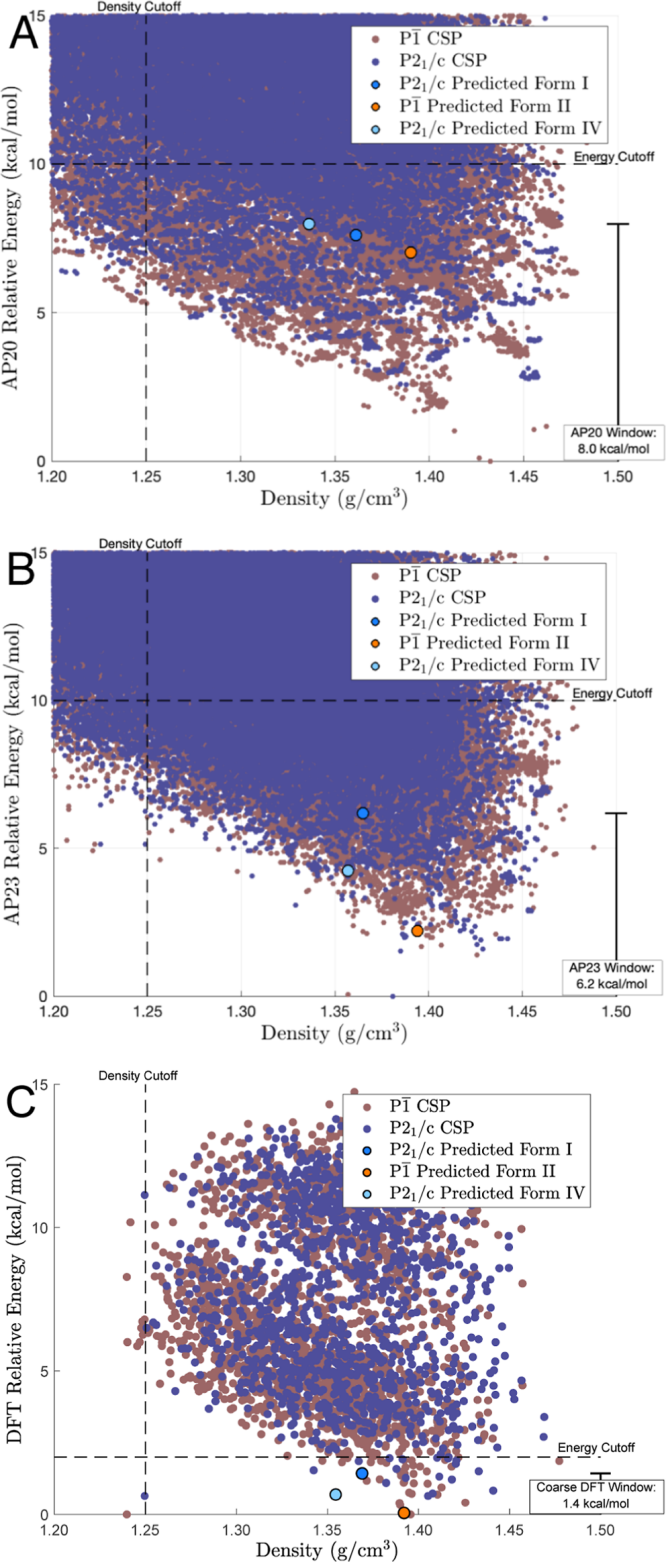
All *NPT* snapshots for the two observed experimental space groups are plotted (*P*1 and *P*2_1_/*c*). The dotted black lines denote cutoffs that prioritize high density (>1.25 g/cm^3^) and low potential energy (<10 kcal/mol). Panel (A) depicts the predicted structures based on minimization using AP20 parameters, whereas panel (B) was minimized with AP23 parameters. Panel (C) shows predictions after minimization with “coarse” DFT. A retrospective energy window is labeled on the right-hand side in each plot that corresponds to a relative energy that preserves the predicted polymorphs that match the experiment.

**Table 4:** Progressive Reduction of Polymorph Candidates Begins from Snapshots Generated during the *NPT* Alchemical Search to the Final DFT Lattice Energy and Density Assessment^a^

space group	*NPT* snapshots	energy and density cutoffs	packing similarity	coarse DFT	precise DFT
*P*1̅	84,122	12,087	6846	1925	27
*P*2_1_/c	87,809	5473	2349	1253	15
total	171,931	17,560	9195	3178	42

a See the text for a complete description.

**Figure 3:**
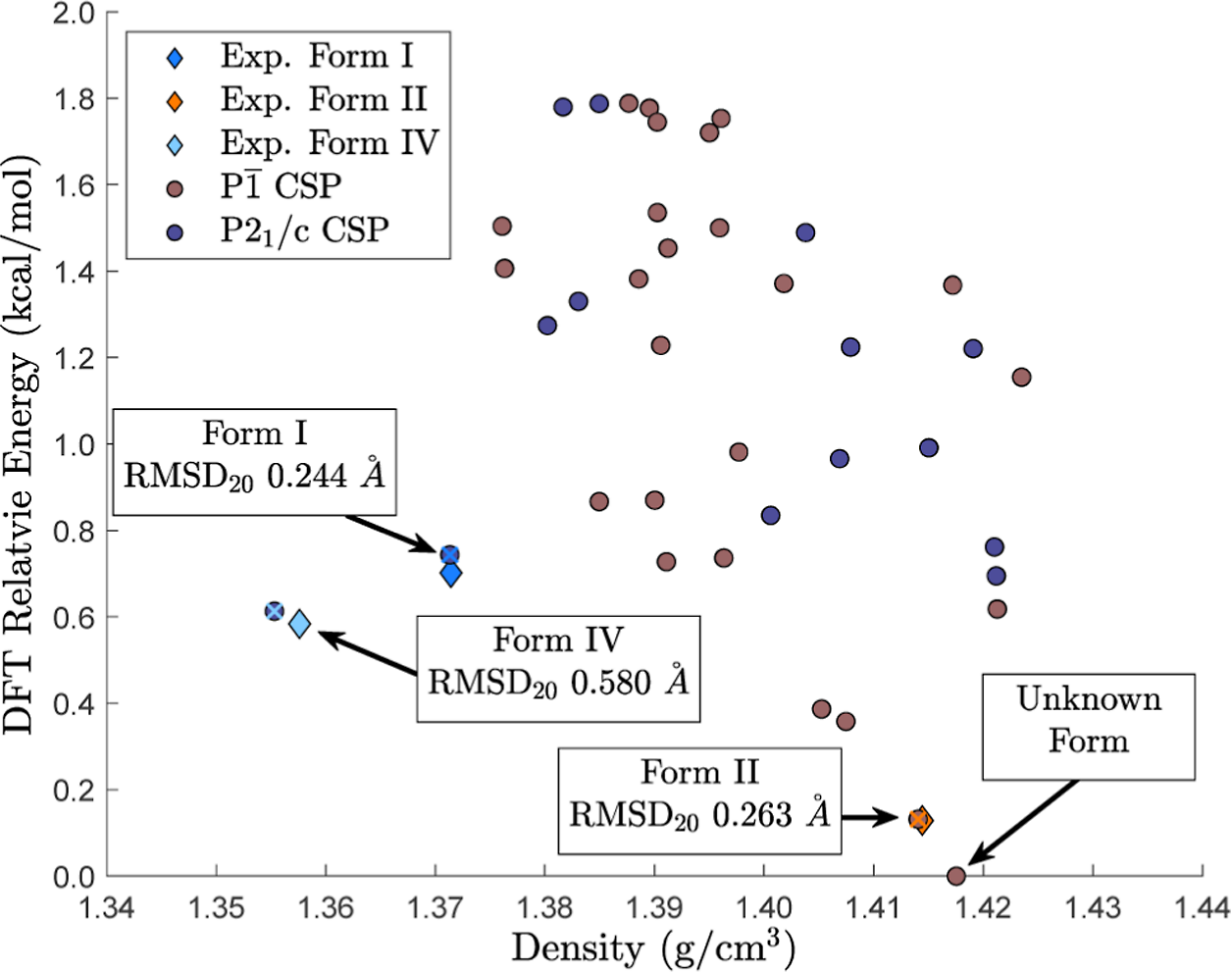
Shown is the density vs “precise” relative DFT-D potential energy for polymorphs discovered via the alchemical *NPT* pipeline and those determined experimentally. Predicted structures that match the experiment are labeled with an arrow along with the rmsd_20_ for their respective experimental polymorph.

**Figure 4:**
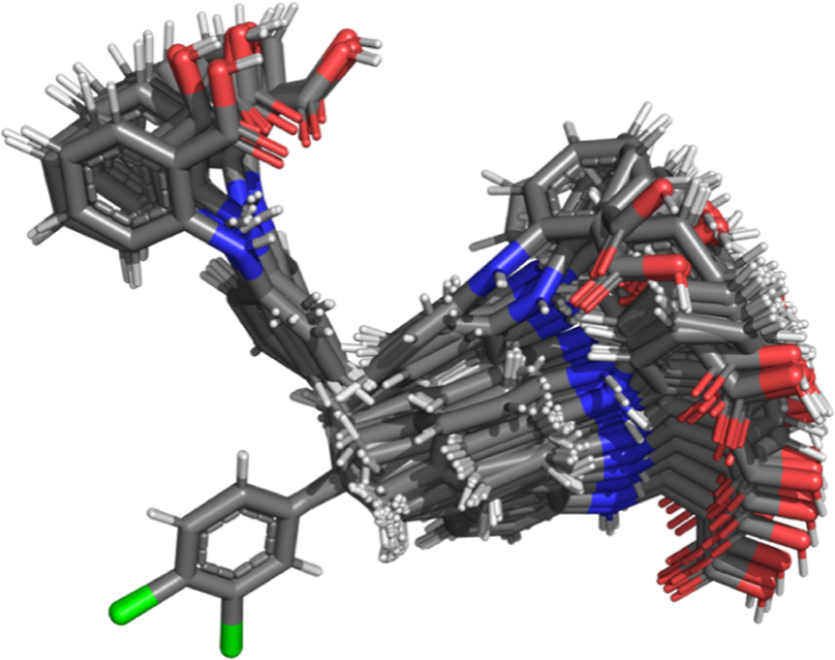
Dichlorobenzene group of compound XXIII was superposed for each saved snapshot to assess the dihedral angles sampled during the CSP procedure.

**Figure 5:**
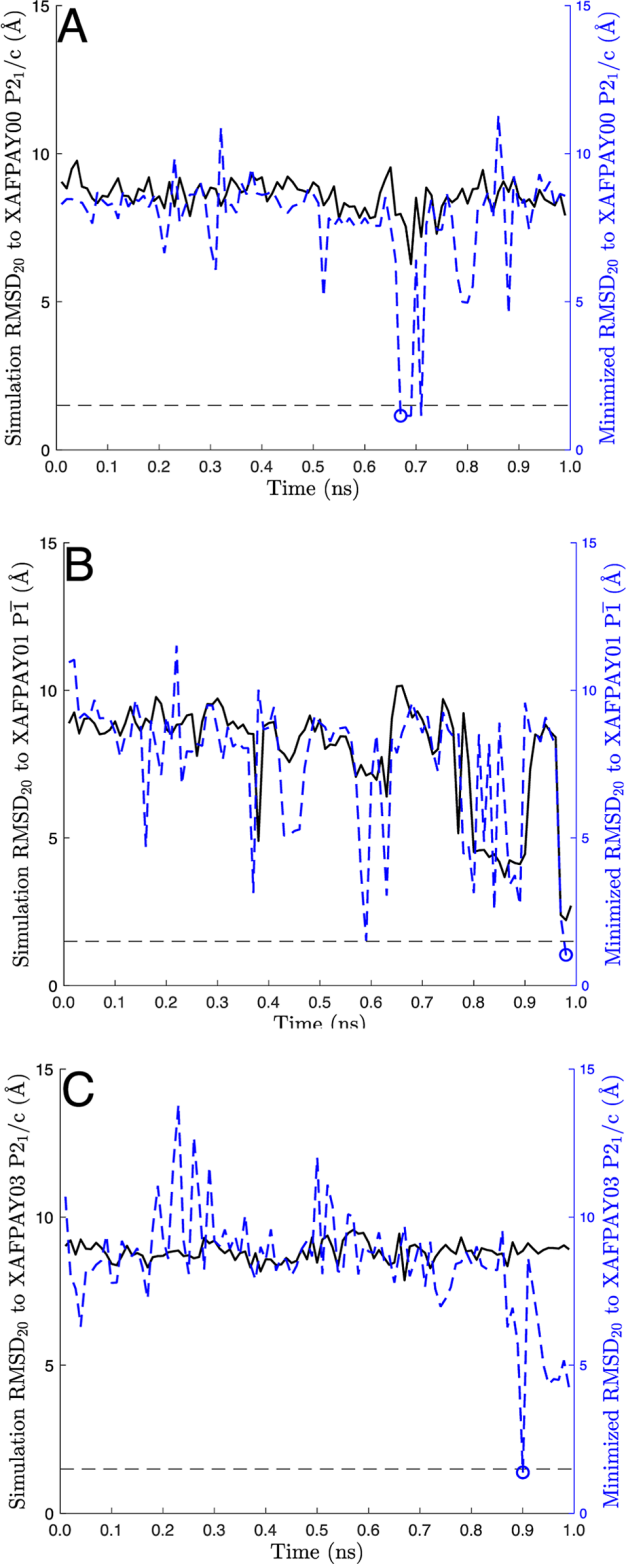
PAC rmsd_20_ values for comparing simulation snapshots to experiments during the AP20 simulations that generated crystal packings close to experiment: (A) XAFPAY with space group *P*2_1_/*c*, (B) XAFPAY01 with space group *P*1, and (C) XAFPAY03 with space group *P*2_1_/*c*. The rmsd_20_ for unminimized structures (*i.e.*, molecular dynamics snapshots) are represented by a solid black line, whereas the rmsd_20_ after crystal minimization is displayed by a blue dashed line. Snapshots that were eventually promoted to DFT minimization are highlighted with a blue circle.

**Figure 6:**
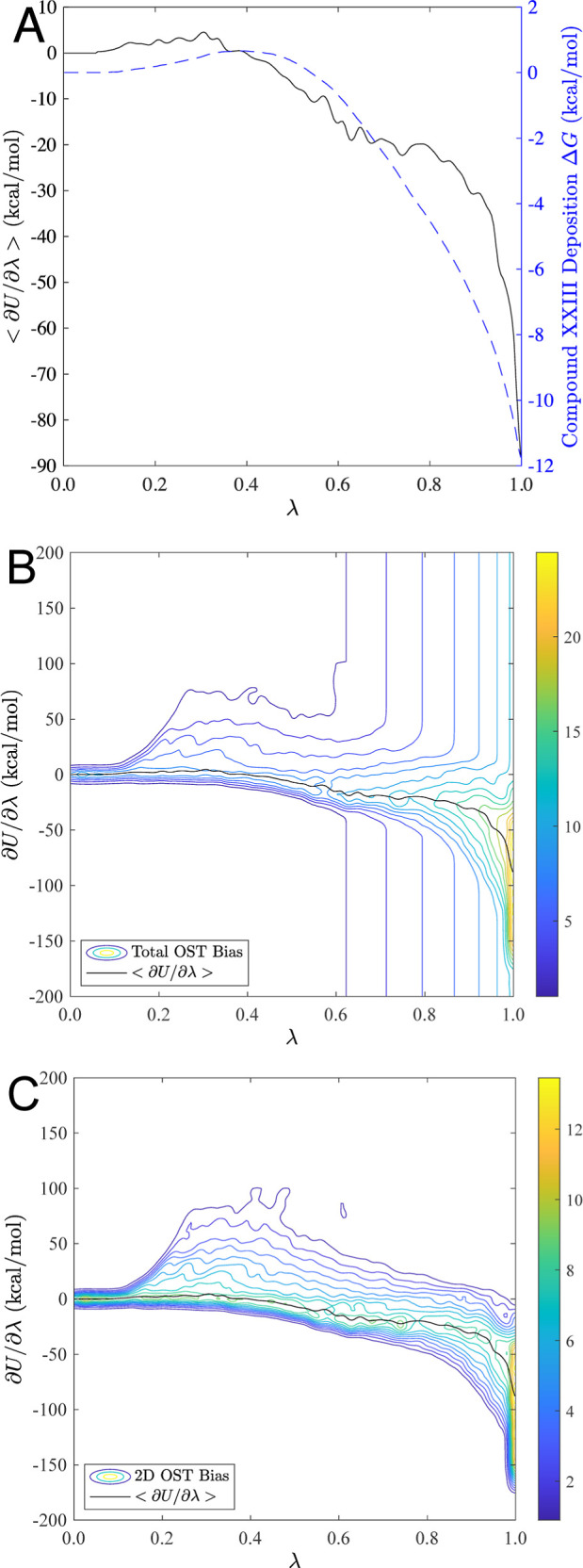
Plots illustrating the OST sampling approach using the compound XXIII simulation that produced the lowest potential energy structure (based on “precise” DFT-D). Panel (A) denotes the ensemble average partial derivative of the potential energy with respect to λ (given by ⟨∂*U*/∂λ⟩) and its integration over the phase transition path to yield the deposition free energy difference. Panels (B,C) are contour plots of the total OST bias and only the 2D component of the bias, respectively.

**Figure 7:**
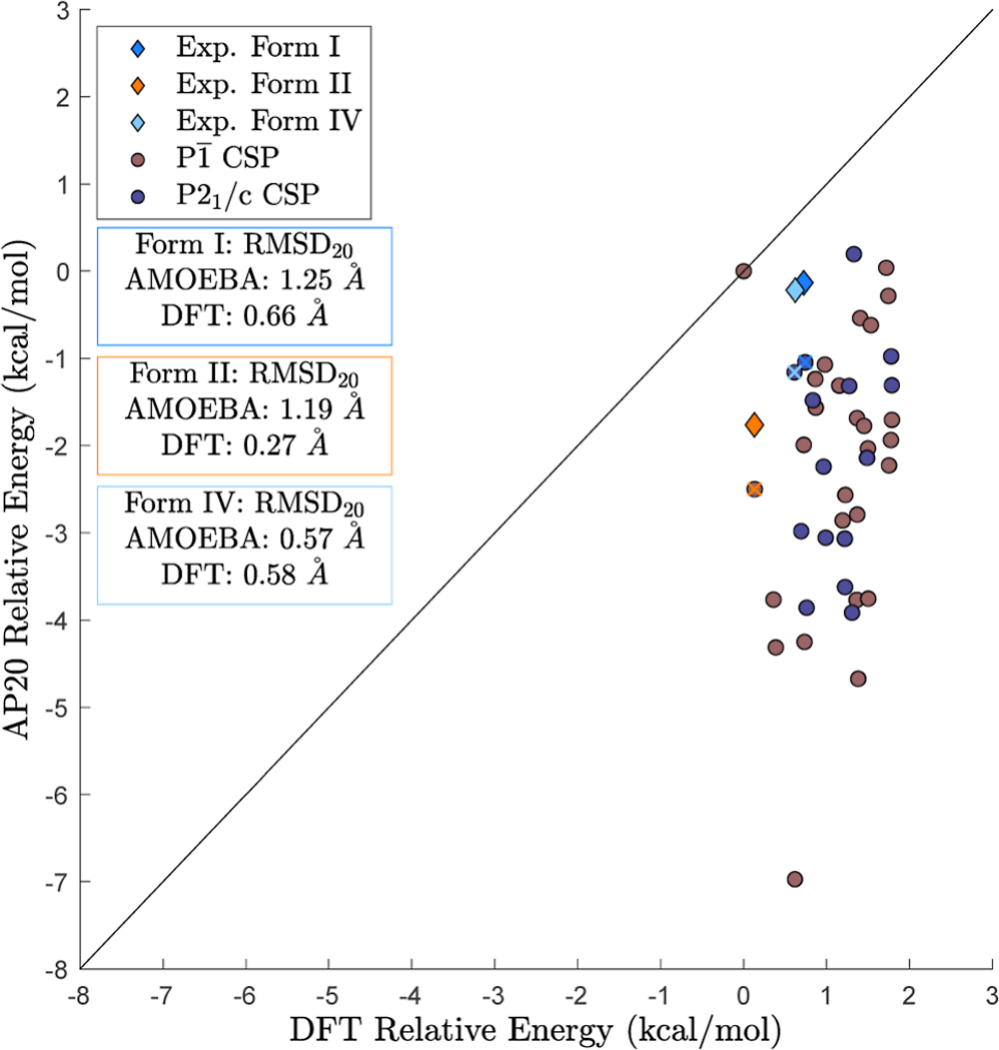
Predicted structures that obtained the lowest relative energy based on “precise” DFT-D are compared to the AP20 relative energies. The diamonds represent the experimental polymorphs, while the circles are structures from the alchemical *NPT* search. Predicted structures with the lowest rmsd_20_ to each experimental polymorph are emphasized with an ‘X’ that is the same color as the experimental polymorph.

**Table 5:** Relative Lattice Energies for the Experimental Structures of Compound XXIII Were Minimized Using a Variety of Models^a^

model	method	form				
		A	B	C	D	E
team 3: Day *et al.*	multipoles and exp-6	0.3	1.3	0.0	0.6	0.1
team 5: van Eijck	charges and exp-6	1.0	0.0	1.3	1.3	1.1
team 14: Neumann *et al*.	PBE + Neumann–Perrin	0.9	0.0	0.0	0.7	0.5
team 18: Price *et al*.	multipoles and exp-6	2.3	0.0	0.8	2.2	1.3
AP20	no polarization	2.4	0.0	2.4	1.3	1.4
	direct	1.7	0.1	1.5	0.9	0.0
	mutual	1.6	0.0	1.4	1.6	0.1
AP23	no polarization	1.7	0.0	2.1	1.8	2.5
	direct	1.0	0.0	1.5	1.2	1.4
	mutual	1.3	0.0	1.8	1.3	1.8

a Rows labeled by “team” are from Table S12 of the sixth blind test of crystal structure prediction. Rows labeled AP20 and AP23 use AMOEBA with the given polarization method (see the text for details). All values are in kcal/mol per molecule.

**Figure 8:**
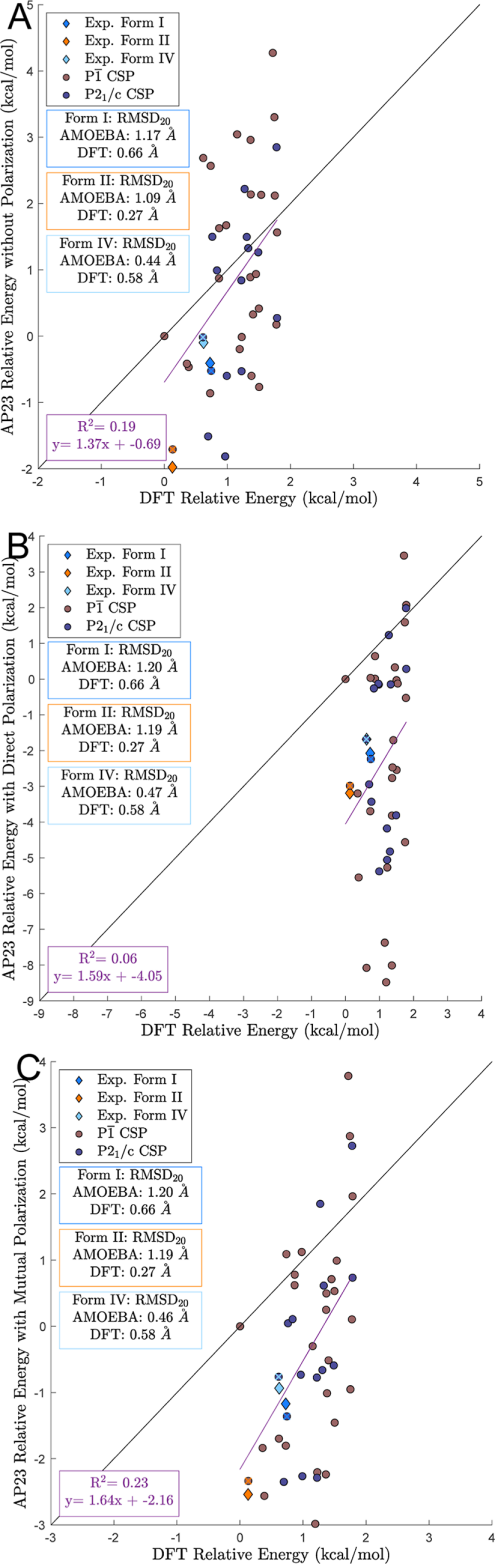
Polymorphs with a relative lattice energy based on “precise” DFT-D within 2 kcal/mol of the lowest energy structure were reminimized with the AMOEBA Poltype2 2023 parameters under three polarization models: (A) no polarization. (B) Direct polarization. (C) Mutual polarization.

### 2.10 Polymorph Ranks Using Density Functional Theory

Final polymorph rankings are typically based on the use of DFT lattice energies, which exceed the accuracy of advanced classical models such as AMOEBA. Here, DFT is leveraged to further optimize both the coordinates and lattice parameters obtained by the alchemical search procedure. In the context of compound XXIII, this also facilitates a retrospective assessment of the AMOEBA “energy window” that defines snapshots that must be promoted to DFT evaluation (i.e., relative to the lowest energy AMOEBA snapshot discovered, what energy range contains observed experimental polymorphs). In this work, the Quantum Espresso (QE) software package was utilized to minimize selected structures.^74^ Structures can be saved in QE format using the FFX command *ExportQE*. The DFT calculations used in this work followed a previously described framework for plane-wave DFT-D optimization of pharmaceutical compounds.^75^ In this work, a two-step minimization procedure with “coarse” and “precise” DFT parameters was used. The coarse minimization utilized 2 *K*-points in each dimension with Grimme-D2, while the precise minimization utilized 3 *K*-points in each dimension with Grimme-D3 (zero damping). Structures within 2 kcal/mol of the lowest energy found by the coarse minimizations were clustered based on a PAC rmsd_20_ of 1.0 Å prior to promotion to the precise optimization. The cutoff of 2 kcal/mol was based on previous findings that showed that experimental polymorphs did not arise from “precise” DFT optimization of higher energy polymorphs.

### 2.11 Criteria for a Successful Prediction

Although the computational efficiency of the AMOEBA force field is essential for the alchemical polymorph search, its accuracy for lattice energies compared with modern periodic DFT methods that include a dispersion correction has not been thoroughly explored. Experimental polymorphs for compound XXIII were obtained from the CCDC and compared to the final predicted polymorphs from the “precise” DFT minimization procedure using the PAC algorithm with molecular clusters of 20 molecules (rmsd_20_). An rmsd_20_ of 1.0 Å or less between the predicted polymorph and the experiment was considered a success. For AMOEBA-minimized structures with an rmsd_20_ of 1.5 Å or less compared to the experiment, downstream DFT-D minimization resulted in rmsd_20_ values below 1.0 Å. This observation is based on the data summarized in Table S1, which shows that predicted structures starting from an AMOEBA rmsd_20_ to experiment of ≤1.5 Å and were promoted to DFT minimization ultimately achieved an rmsd_20_ to experiment of ≤1.0 Å. The 1.0 Å cutoff for the final similarity ranking was chosen based on crystal structure similarity metrics used previously.^11^ These cutoffs can be adjusted depending on the system (e.g., larger APIs where more variance is observed but maintain similar crystal packing).

## 3 Results

### 3.1 Number of Crystal Packings: From Alchemical *NPT* to Final DFT Rankings

Figure 2 shows the resulting prioritization of snapshots based on the combination of a sufficiently high density (>1.25 g/cm^3^) and low potential energy (within 10 kcal/mol of the most stable observed structure). Figure S2 shows predictions from all 15 space groups that were utilized in this work.

In Table 4, the progressive reduction in the number of polymorph candidates is illustrated for space groups *P*1̅ and *P*2_1_/**c** by using the overall procedure described above. The alchemical *NPT* search yielded 84,122 and 87,809 structures for *P*1̅ and *P*2_1_/**c**, respectively. This was reduced to 6846 and 5473 using conservative energy (<10 kcal/mol) and density (>1.25 g/cm^3^) cutoffs, followed by further reductions to 2683 and 2349 structures using the PAC crystal packing similarity metric with iterative hierarchical clustering, respectively. Finally, “coarse” and “precise” DFT optimizations produced the final collection of 27 and 15 possible polymorphs for *P*1̅ an *P*2_1_/*c*, respectively. Further assessment is described below in terms of DFT lattice energy, density, and crystal packing similarity to experiment.

In the sixth blind CSP competition, between ∼146,000 and 400,000 CPU hours were utilized to predict all the three *Z*′ = 1 polymorphs of compound XXIII. In this work, the *NPT* search over the top 43 space groups would utilize roughly 21,000 CPU hours, while the “coarse” and “precise” DFT calculations would utilize around 297,000 and 342,000 CPU hours, respectively, for a total of ∼660,000 CPU hours. Shifting the energy window from 10 kcal/mol down to 9 kcal/mol reduced the number of promoted structures by roughly 33% and would reduce cost to approximately 198,000 and 228,000 CPU hours for coarse and precise DFT minimizations, respectively. Finally, use of an 8 kcal/mol window, which is appropriate for the AP23 parameters (see Figure 2B), removes 45% of the included structures and thereby requires 134,000 and 154,000 CPU hours for the coarse and precise DFT calculations, respectively.

### 3.2 DFT Lattice Energy, Density, and Crystal Packing Assessment

Shown in Figure 3 is a plot of the density vs potential energy (computed using “precise” DFT parameters following minimization) for predicted polymorphs compared to experimentally observed polymorphs. Predictions that concord with the experiment are emphasized with an arrow, and the PAC rmsd_20_ is included to quantify the deviation of the predicted polymorph from the experiment. All the three experimental polymorphs that contain a single molecule in the asymmetric unit were located. One experimentally unobserved polymorph with lower potential energy and higher density than the experimental polymorphs was discovered. Further simulations could be used to evaluate the free energy difference between polymorphs and investigate if any polymorphs reside in the same free energy minimum.

The addition of the MC barostat to the FFX deposition/sublimation alchemical simulations allows all degrees of freedom to be sampled, including lattice parameters, rigid body rotations and translations, and finally intramolecular degrees of freedom, such as torsional angles. Torsional sampling can be observed by overlapping the dichlorobenzene ring of all predicted conformers as shown in Figure 4. All conformations maintained the intramolecular hydrogen bond between the amine and carboxylic acid groups, indicating that rotation around the torsion between the amino and benzoic acid groups is on a longer time scale than that employed in this work. To explore this degree of freedom (or other torsional degrees of freedom with large energy barriers), it is possible to employ more sampling per walker, a larger OST bias, and/or utilization of multiple starting conformations.

One avenue to the assessment of convergence is locating low-energy snapshots multiple times. We compared each of the AMOEBA-minimized snapshots to the experimental polymorphs by computing the rmsd_20_ with PAC. Many of the predicted structures that were similar to experimental polymorphs were filtered out during the clustering step based on the PAC rmsd_20_ cutoff of 1.5 Å, which further supports use of PAC to quantify crystal packing similarity (i.e., of the seven predicted structures in Table S1 that were similar to the experimental *P*1̅ polymorph, only one remained after cluster-based filtering). Post hoc analysis demonstrated that 6 of the 6000 simulations successfully found the experimentally determined *P*1̅ polymorph and three *NPT* walkers found the structures for *P*2_1_/*c* (two found form A and one found form D). Shown in Figure 5 is the rmsd_20_ as a function of time (snapshots were saved every 10 ps) for three selected walkers that located an experimental polymorph.

The OST method employed by this CSP algorithm constructs a time-dependent bias via application of a series of 2D Gaussian hills, as described above. In Figure 6A, the ensemble average partial derivative of the potential energy with respect to the path variable λ (given by ⟨∂*U*/∂λ⟩) is plotted as a solid black line. The blue dashed line represents the deposition free energy difference at λ relative to that in the vacuum state (λ = 0). Figure 6B is a contour plot of the total OST bias (i.e., the sum of the 1D and 2D bias contributions). In the crystalline phase (near or at λ = 1), the OST bias facilitates exploration of the crystal free energy landscape away from the free energy minimum, which promotes crossing free energy barriers in the so-called orthogonal direction (i.e., along ∂*U*/∂λ). Figure 6C is a contour plot of only the 2D OST bias, which serves to isolate the bias along ∂*U*/∂λ by removing the 1D bias (a constant for any fixed value of λ). Overall, the goal for the OST bias was to achieve a random walk along λ while also accelerating the crystal packing search within approximately 10 kcal/mol of the basin(s) representing the crystalline free energy minima. In this way, it is possible for simulations that locate a local free energy minimum to escape over local barriers and to discover more favorable polymorphs nearby.

For the retrospective analysis of the AP20 parameters used for the alchemical *NPT* search algorithm, all structures within 2 kcal/mol of the lowest energy structure (based on “precise” DFT-D) were minimized using the AP20 force field as depicted in Figure 7. This resulted in nearly a 10 kcal/mol AP20 energy range. One snapshot minimized with the AP20 parameters was 2.3 kcal/mol more stable than any other structure (including those minimized starting from experimental coordinates). Investigation of this outlier revealed that the cause was primarily the positive monopole parameters for the chlorine atoms defined by the AP20 parameters, which motivated updating of the AP23 parameters. Functional groups that are not present in the AMOEBA small organic molecule, protein, or nucleic acid force fields have generally been studied less by the community and might also be expected to improve over time.

In Table 5, the AP20 and AP23 relative lattice energies are compared to those calculated by groups in the sixth blind CSP contest^76^ to further assess both parameter sets. Furthermore, the impact of different treatments of polarization was assessed due to its influence on the computational efficiency. Three polarization models were considered: (A) no polarization such that electrostatics are based only on permanent atomic multipoles, (B) direct polarization whereby induced dipoles are a function of the permanent atomic multipole field, but do not influence each other, and (C) mutual polarization based on a self-consistent field calculation (i.e., the full AMOEBA model). Most groups (including the AMOEBA results) determined that form B was the most stable polymorph by around 1 kcal/mol. These results support the idea that both the AP20 and AP23 AMOEBA models (with different levels of polarization) are somewhat comparable in quality to those used in prior work to model the experimental polymorphs of compound XXIII.

Search structures that were minimized with 2 kcal/mol of the lowest energy polymorphs were reminimized using the AP23 parameters without polarization, with direct polarization, and with mutual polarization to produce Figure 8A–C, respectively. Minimizations without polarization and with mutual polarization resulted in energy ranges of around 6 kcal/mol, as depicted in Figure 8A,C, respectively. However, use of direct polarization resulted in an energy range of more than 10 kcal/mol. Thus, direct polarization degrades the accuracy of compound XXIII relative lattice energies and increases the likelihood of favorable structures being erroneously filtered out prior to DFT assessment (unless a large energy window is employed). For this reason, utilization of relative lattice energies without polarization is more promising than use of direct polarization for both the alchemical *NPT* search and AMOEBA minimizations, at least in the context of compound XXIII. However, absolute lattice energies for the experimental polymorphs (Table S3) demonstrate the expected trend, whereby direct polarization approached the lattice energy of mutual polarization more closely than no polarization. Further investigation is needed to fully understand the limitations of “direct” polarization. For example, it is possible that anisotropic atomic polarizability tensors could improve performance for both direct and mutual polarization.

## 4 Conclusions

We have described an open-source crystal polymorph search algorithm that efficiently samples from the *NPT* ensemble by extending a previously established *NVT* deposition/sublimation alchemical path. Simulation within the *NPT* ensemble during the CSP search incorporates temperature and pressure effects that were previously neglected during a search of the crystal potential energy landscape. Following the *NPT* search, the pipeline employs energy and density cutoffs, followed by a novel clustering approach to remove duplicate crystal packings. The retrospective prediction of experimental polymorphs for several organic molecules has been achieved,^54^ and the overall approach was presented in detail for compound XXIII here.

Furthermore, our pipeline supports a variety of potential energy functions in addition to AMOEBA, including both OPLS-AA^26^ and OpenFF.^25^ Both the accuracy and efficiency of a given search protocol depend on the chosen force field. As accuracy decreases, larger energy cutoffs are needed to ensure that experimentally observable polymorphs are kept throughout the pipeline and promoted to assessment by DFT. For this reason, further improvements to the accuracy of polarizable atomic multipole force fields (e.g., AMOEBA+^44^ and HIPPO^77^) are expected to improve overall efficiency (i.e., fewer snapshots will need to be evaluated using DFT-D). The conformational space of the API also impacts the efficiency of the search because molecular dynamics simulations can be impeded by energy barriers. For example, convergence of the search can be slowed by the need to sample torsional barriers, by the addition of multiple molecules within the asymmetric unit, or when transitioning to larger and more flexible molecules. To mitigate energy barriers, the OST sampling method has been employed.

Future work will incorporate accurate evaluation of free energy differences between polymorphs to rank crystal packing candidates and help to reduce reliance on DFT-D calculations.^21^ Further investigation is also required to determine the total amount of *NPT* simulation required to converge sampling for ever larger and/or more flexible molecules. Nevertheless, the efficiency of using classical models (or neural network potentials) to sample an alchemical path between vacuum and crystalline phases lends itself to larger systems where neglecting entropic effects is increasingly a poor approximation. For this reason, the approach presented here is promising for predicting polymorphs composed of peptides or other complex polymers.

## Supporting Information Available

CSP pipeline overview, optimal CSP polymorphs, statistics for all predicted space groups, polarization comparisons, scaled axes, PAC clusters, DFT specifications, and *NVT* deposition free energies based on OST (PDF)
Example parameters and structures produced by the CSP algorithm (ZIP)

## Abbreviations

AMOEBA: Atomic Multipole Optimized Energetics for Biomolecular Applications
AP20: AMOEBA parameters from 2020
AP23: AMOEBA parameters from 2023
API: active pharmaceutical ingredient
BFGS: Broyden–Fletcher–Goldfarb–Shanno algorithm
CCDC: Cambridge Crystallography Data Centre
CGenFF: CHARMM general force field
CPU: central processing unit
CSD: Cambridge Structural Database
CSP: crystal structure prediction
DFT: density functional theory
DMA: distributed multipole analysis
FFX: Force Field X
GAFF: general Amber force field
GB: gigabyte
GDMA: Gaussian DMA
GE: generalized ensemble
MC: Monte Carlo
MM: molecular mechanics
*NPT*: isobaric–isothermal ensemble
*NVT*: canonical ensemble
OPLS-AA: optimized potentials for liquid simulations all atoms
OST: orthogonal space tempering
PAC: progressive alignment of crystals
PBE: Perdew–Burke–Ernzerhof exchange and correlation
PME: particle mesh Ewald
QE: Quantum Espresso
QM: quantum mechanical
RAM: random access memory
rmsd_20_: root-mean-square deviation for clusters of 20 molecules
SCF: self-consistent field
