# Supplementary material for: Crystal Polymorph Search in the NPT Ensemble via a Deposition/Sublimation Alchemical Path
Source: Cryst Growth Des. 2024 Mar 9;24(8):3205–17. doi: 10.1021/acs.cgd.3c01358 (PMC11036363; doi:10.1021/acs.cgd.3c01358)
Supplement: Supplementary file 1 — cg3c01358_si_001.pdf [file cg3c01358_si_001.pdf]

Supplemental Information for:

# Crystal Polymorph Search in the NPT Ensemble via a Deposition/Sublimation Alchemical Path

Aaron Nessler (ORCID: 0009-0004-2502-6204)[1], Okimasa Okada[2], Yuya Kinoshita[3], Koki Nishimura[3], Hiroomi Nagata[4], Kaori Fukuzawa (ORCID: 0000-0001-5357-8250) [5], Etsuo Yonemochi (ORCID: 0000-0001-5255-5129)[6], and Michael J. Schnieders (ORCID: 0000-0003-1260-4592)[1,7,*]

[1]University of Iowa Department of Biomedical Engineering, 103 South Capitol Street, 5601 Seamans Center for the Engineering Arts and Sciences, Iowa City, IA, 52242

[2]Sohyaku. Innovative Research Division, Mitsubishi Tanabe Pharma Corporation, Japan

[3]Analytical Development, Pharmaceutical Sciences, Takeda Pharmaceutical Company Limited, 2-26-1, Muraoka-Higashi, Fujisawa, Kanagawa 251-8555, Japan

[4]CMC Modality Technology Laboratories, Production Technology and Supply Chain Management Division, Mitsubishi Tanabe Pharma Corporation, Japan

[5]Graduate School of Pharmaceutical Sciences, Osaka University, 1-6 Yamadaoka, Suita, Osaka 565-0871, Japan

[6]Department of Physical Chemistry, School of Pharmacy and Pharmaceutical Sciences, Hoshi University, 2-4-41 Ebara, Shinagawa-ku, Tokyo 142-8501, Japan

[7]University of Iowa Department of Biochemistry, 51 Newton Road, 4-403 Bowen Science Building, Iowa City, IA, 52242

*Corresponding Author: michael-schnieders@uiowa.edu

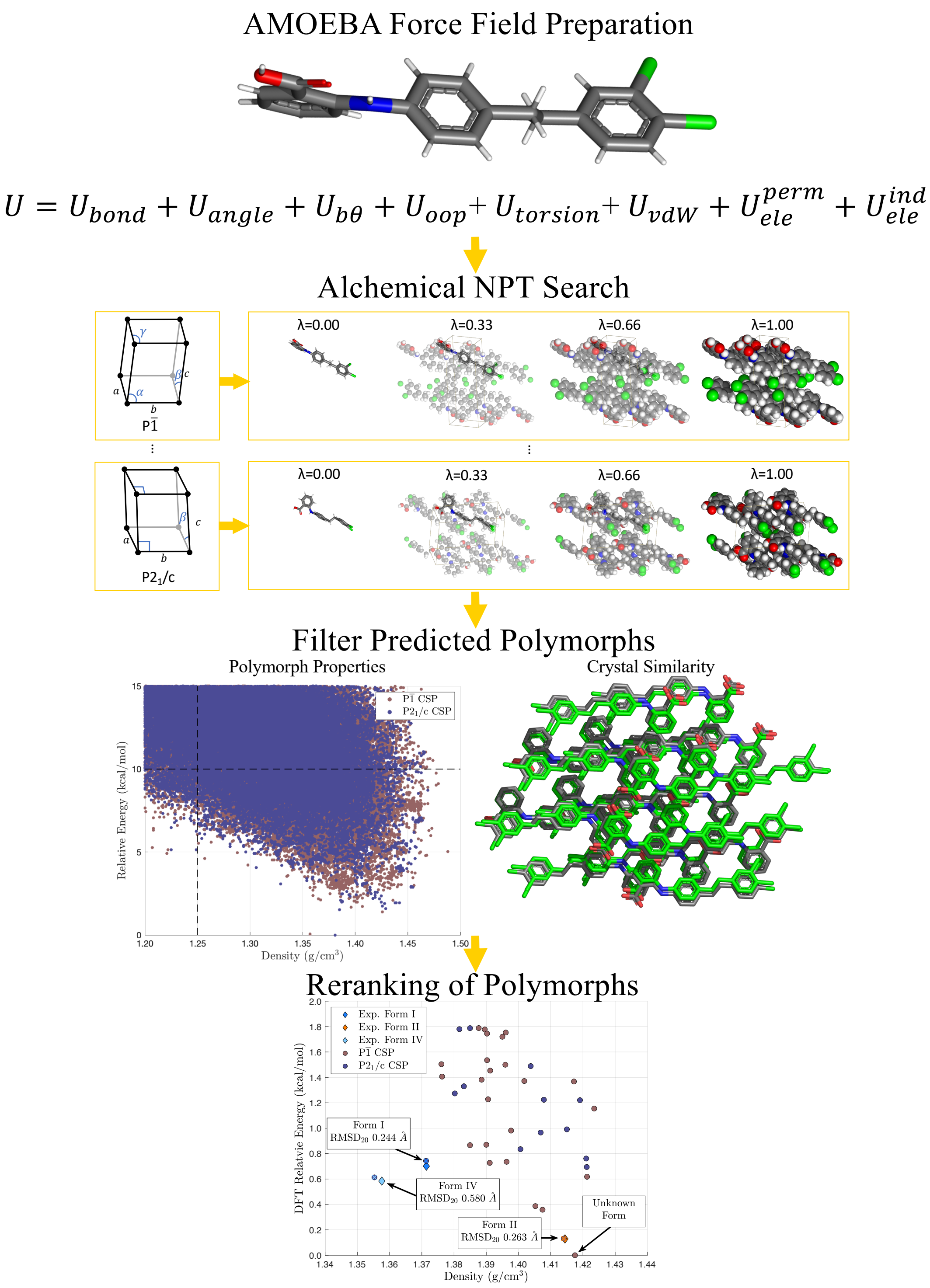


SI Figure 1. A diagram describing the steps used to predict the crystal polymorphs. After parameterization, the polymorph search method generates space groups for the input structure. After the thermodynamics simulation in the NPT ensemble, each snapshot is minimized and then filtered (*i.e.*, using potential energy and density). Similar structures are then removed using the PAC algorithm. Finally, the polymorphs are reranked with a more accurate method such as DFT-D.

SI Table 1. Predicted polymorphs with $RSMD_{20}$ to experiment less than 1.5 Å were followed through the pipeline. Entries with a minus sign were filtered out (*e.g.*, due to energy/density cutoffs or PAC similarity clustering). The entry highlighted in blue does not match an experimental polymorph, but its energy and density are competitive with observed structures.

| Space Group | Run | Job | Structure | $RMSD_{20}$ PAC (Å) | Relative Energy (kcal/mol) | Density QM (g/cm$^3$) | Relative Energy (kcal/mol) | Density QM (g/cm$^3$) | $RMSD_{20}$ QM (Å) |
|---|---|---|---|---|---|---|---|---|---|
| $P\bar{1}$ | 2 | 93 | 137 | 1.13 | 0.894 | 1.46 | - | | |
| | 2 | 55 | 36 | 1.15 | 0.078 | 1.48 | - | | |
| | 3 | 49 | 80 | 1.19 | -0.780 | 1.49 | 0.13 | 1.41 | 0.27 |
| | 3 | 49 | 79 | 1.16 | - | | | | |
| | 3 | 10 | 131 | 1.15 | -0.222 | 1.49 | - | | |
| | 4 | 82 | 58 | 1.12 | -0.515 | 1.50 | - | | |
| | 5 | 32 | 106 | 1.12 | - | | | | |
| | 1 | 09 | 193 | 8.58 | 0.000 | 1.45 | 0.00 | 1.42 | 8.12 |

| Space Group | Run | Job | Structure | $RMSD_{20}$ PAC (Å) | Relative Energy (kcal/mol) | Density QM (g/cm$^3$) | Relative Energy (kcal/mol) | Density QM (g/cm$^3$) | $RMSD_{20}$ QM (Å) | Form |
|---|---|---|---|---|---|---|---|---|---|---|
| $P2_1/c$ | 2 | 81 | 82 | 1.25 | 0.00 | 1.45 | 0.00 | 1.37 | 0.66 | A |
| | 2 | 81 | 83 | 1.24 | - | | | | | A |
| | 5 | 79 | 94 | 0.78 | 1.12 | 1.38 | - | | | D |
| | 5 | 79 | 98 | 0.57 | 0.02 | 1.41 | -0.74 | 1.35 | 0.58 | D |
| | 5 | 79 | 99 | 0.60 | 0.54 | 1.39 | - | | | D |
| | 5 | 34 | 45 | 1.17 | 0.06 | 1.43 | - | | | A |
| | 5 | 34 | 49 | 1.17 | - | | | | | A |
| | 5 | 34 | 50 | 1.17 | - | | | | | A |

SI Table 2: Quantification of the number of structures predicted by the NPT search for each space group, followed by the number of structures that met the energy (within 10.0 kcal/mol of the most stable structure) and density (>1.25 g/cm$^3$) criteria.

| Space Group | Number of NPT Structures | Number of Structures Remaining |
|---|---|---|
| P1 | 94769 | 1247 |
| $P\bar{1}$ | 84122 | 12087 |
| $P2_1$ | 90796 | 2438 |
| C2 | 86701 | 734 |
| Cc | 97813 | 894 |
| P2/c | 79344 | 1656 |
| $P2_1/c$ | 87809 | 5473 |
| C2/c | 88100 | 2437 |
| $P2_12_12_1$ | 97650 | 2900 |
| $Pca2_1$ | 90390 | 793 |
| $Pna2_1$ | 96818 | 1281 |
| Pbcn | 90747 | 1157 |
| Pbca | 96796 | 2934 |
| Pnma | 70351 | 69 |
| $H\bar{3}$ | 59947 | 253 |
| Total | 1312153 | 36353 |

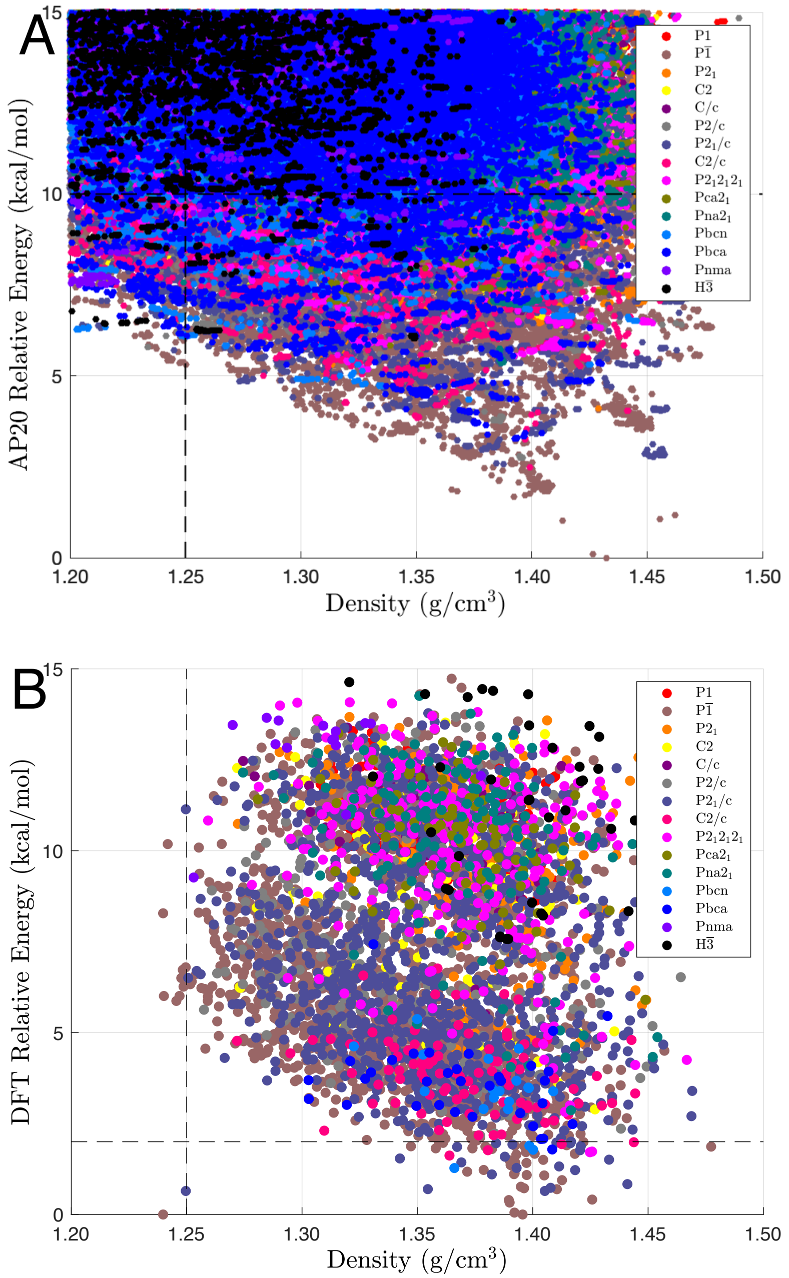


SI Figure 2. NPT snapshots for all 15 examined space groups are plotted. The dotted black lines denote cutoffs to filter polymorphs by prioritizing high density (>1.25 g/cm$^3$) and low potential energy (<10 kcal/mol above the lowest energy polymorph found). Panel A depicts results from the NPT simulations while panel B contains conformers that were reevaluated via "coarse" DFT.

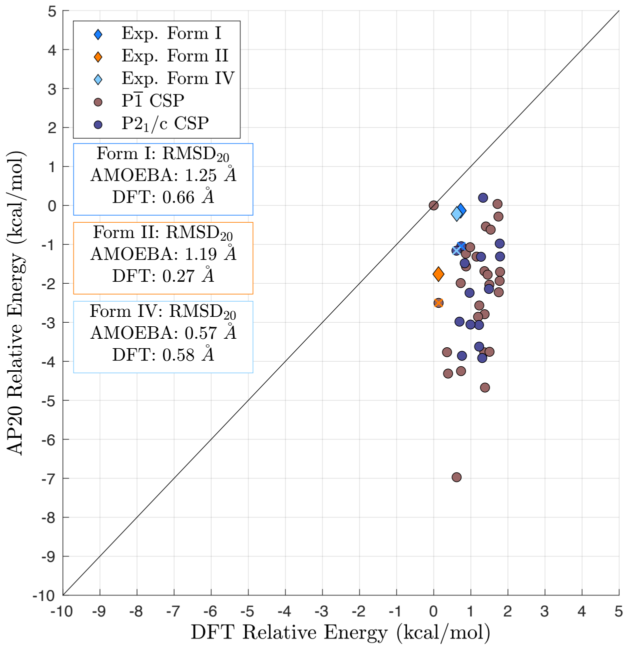


SI Figure 3. The predicted structures with lowest relative energy values based on "precise" DFT are compared with the relative lattice energies of the AMOEBA PolType 2020 parameters. The axes are scaled to facilitate visual comparison to the relative energy values in S.I. Figures 4A-C.

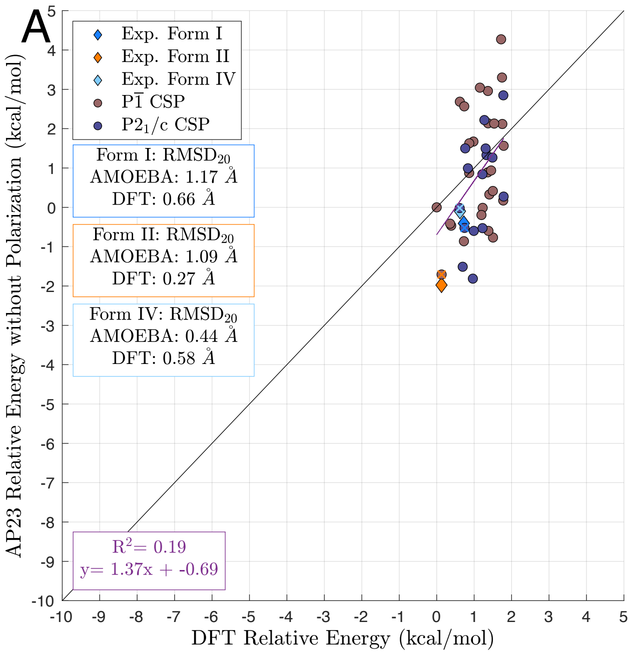

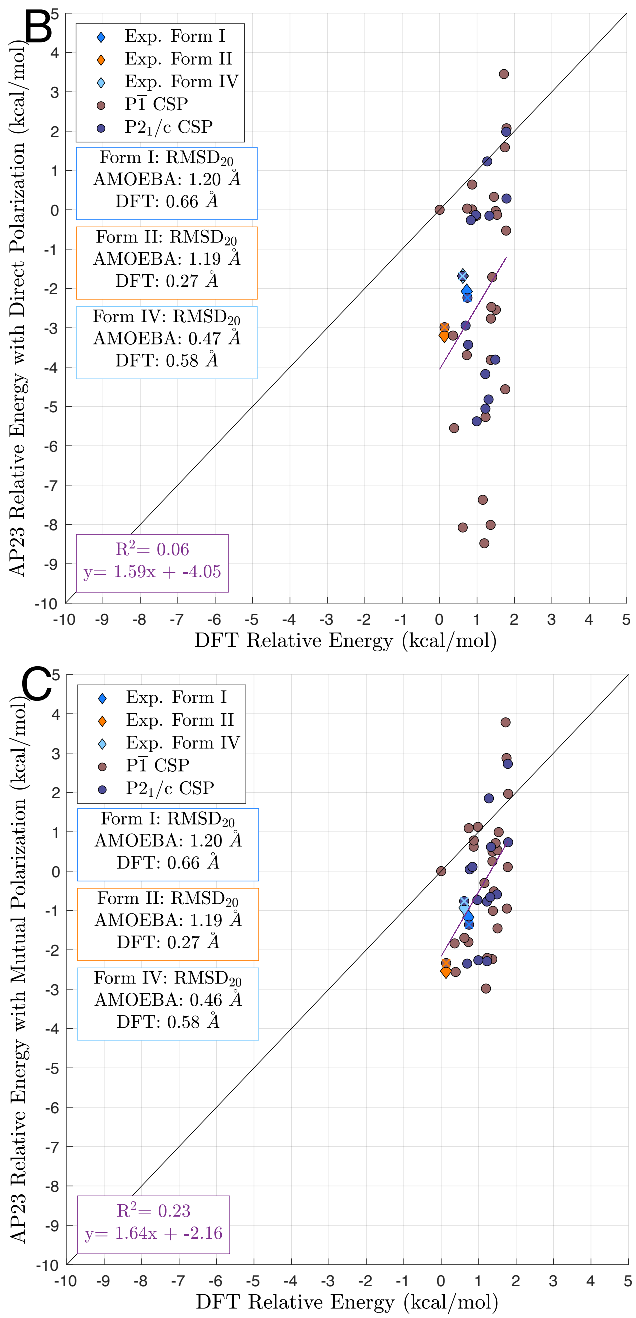


SI Figure 4. Predicted structures with a relative energy within 2 kcal/mol of the lowest energy structure from "precise" DFT were minimized with the AMOEBA Poltype2 2023 parameters with different electrostatic models: A) no polarization, B) direct polarization, and C) mutual polarization. These plots are equivalent to those in Figure 8 A-C, but with fixed x- and y-axis ranges defined by the extent of the data in Panel B. Alternatively, Figure 8 A-C use variable x- and y-axis ranges optimized to their data alone.

## Further Analysis of Electrostatic Models

The coefficient of determination ($R^2$) between each AMOEBA electrostatic model and to "precise" DFT is given in SI Table 3 below. Although the direct polarization model offers a better $R^2$ to the AMOEBA mutual polarization model than does no polarization, the $R^2$ to DFT is worse than no polarization. The modest increase in $R^2$ between no polarization and full mutual polarization to "precise" DFT suggests more sophisticated polarization models (*e.g.,* anisotropic atomic polarizability) may be helpful in the context of ranking organic polymorphs.

SI Table 3. The coefficient of determination between AMOEBA polarization models and to both AMOEBA mutual polarization ($R^2$ from SI Figure 5 below) and "precise" DFT ($R^2$ from Figure 8 in the main text).

| AMOEBA Electrostatics Model | Coefficient of Determination ($R^2$) Mutual | DFT |
|---|---|---|
| No Polarization | 0.53 | 0.19 |
| Direct | 0.67 | 0.06 |
| Mutual | - | 0.23 |

The NPT generated structures that attained an energy within 2 kcal/mol of the lowest energy structure using "precise" DFT-D were minimized with the AP23 parameters using the mutual polarization model. The relative energy of these polymorphs utilizing each electrostatic model are plotted below in SI Figure 5. The relative energy scale is based on subtracting the AP23 minimized energy of the polymorph that attained the lowest "precise" DFT-D energy.

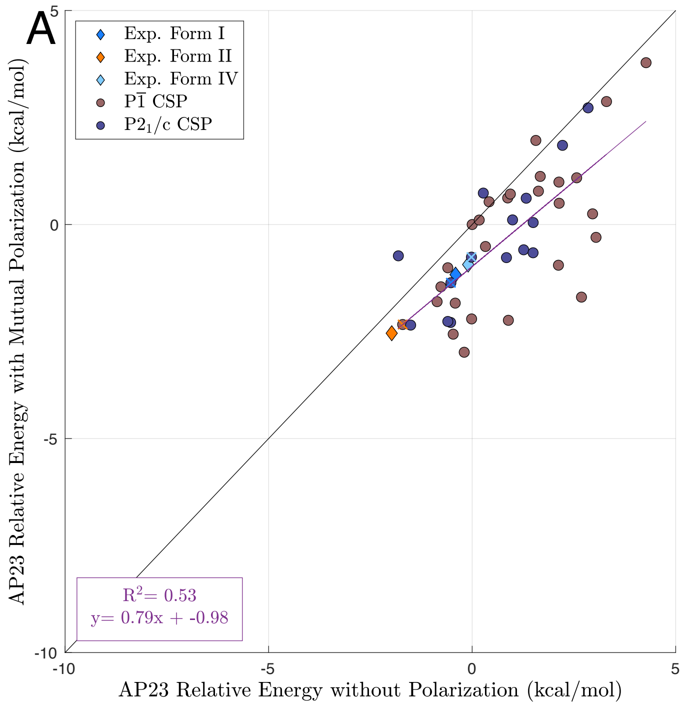

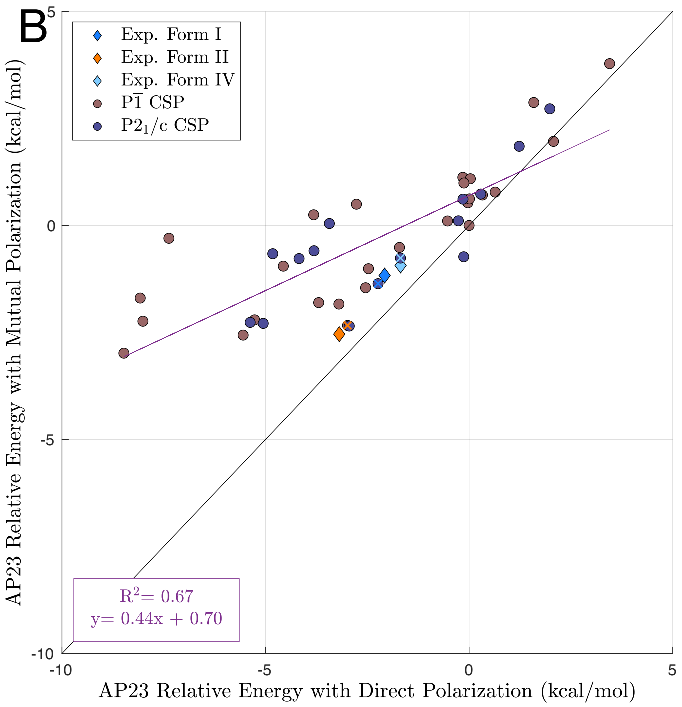


SI Figure 5. Relative polymorph energy values without polarization and with direct polarization are plotted against mutual polarization to quantify the impact of the different electrostatic models. Energies are relative to the AP23 minimized polymorph that attained the lowest "precise" DFT-D energy.

**Visualization of PAC Comparison**

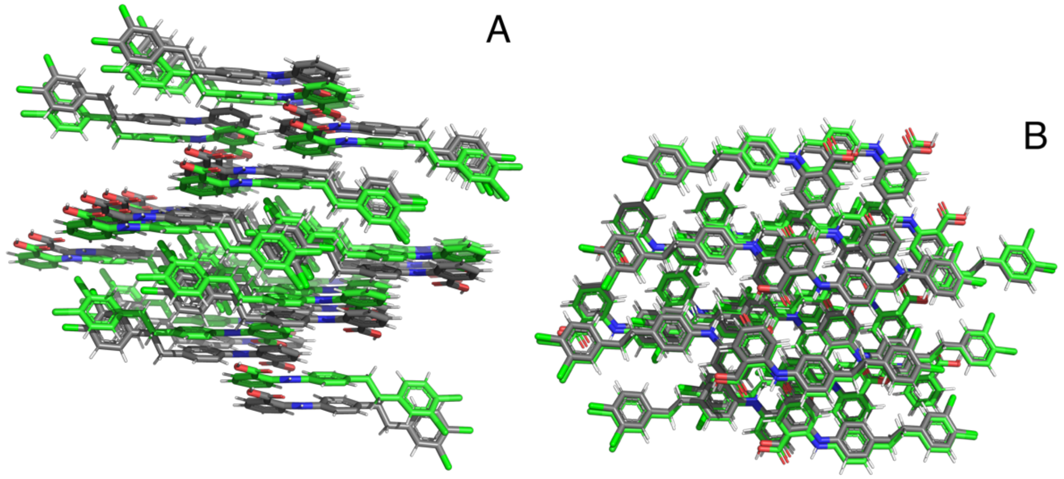


SI Figure 6. Representative comparisons of superimposed clusters of 20 molecules produced by the PAC algorithm are depicted to emphasize the level of similarity that is differentiated with the chosen cutoffs. The superimposed clusters in panel A have an $RMSD_{20}$ of 1.54 Å and the crystal packings they represent are therefore treated as distinct polymorphs for further evaluation. On the other hand, those in panel B have an $RMSD_{20}$ of only 0.38 Å, which allows one copy to be eliminated.

## DFT Specifications

A two-step minimization using the BFGS method (for both ions and cell dynamics) was utilized, where both used 500 steps of variable cell relaxation with a force convergence threshold of $1.0x10^{-2}$ a.u., kinetic energy cutoffs of 55.0 Ry for wavefunctions and 550 Ry for the charge density/potential. Convergence of the electron SCF was enforced to a cutoff of $1.0x10^{-7}$ a.u. Pseudopotentials generated by the Rappe Rabe Kaxiras Joannopoulos method with Perdew-Burke-Ernzerhof exchange and correlation (PBE) were used for all atomic species[69]. The *coarse* minimization was performed on a wide range of predicted structures that had passed previous analysis steps (*i.e.*, energy, density, and similarity filtering) utilizing two k-points in each dimension to represent the Brillouin zone. Additionally, the Grimme-D2 dispersion[70] correction and a convergence criterion of $1.0x10^{-3}$ a.u. were used for this *coarse* minimization. After screening these structures based on their energy from the *coarse* minimization (within 2 kcal/mol of the lowest energy structure) and similarity (based on PAC $RMSD_{20}$ = 1.0 Å), we performed a second *precise* minimization with three k-points in each dimension, Grimme-D3 (zero damping) dispersion correction[71], and a convergence criterion of $1.0x10^{-5}$ a.u.

## Impact of Orthogonal Space Tempering

Two sets of simulations were performed using the NVT ensemble to assess the use of OST. Each contained ten trials of 100 simulations sampling for 10 nsec. Both sets were supplied the experimental lattice parameters to simplify the search procedure to atomic coordinates within a fixed lattice. A random translation and rotation were applied to the molecule in vacuum prior to the alchemical NPT simulation beginning. On average, 0.4% of the simulations without the 2D OST bias reached the experimental structure (*i.e.*, within an $RMSD_{20}$ of 1.5 Å) whereas 0.8% were successful when the 2D OST bias was included used. These results suggest that the 2D OST bias offers a ~2x improvement in efficiency for Compound XXIII when the search is limited to atomic coordinates. Representative results from using OST with the 2D bias are given in SI Figures 7A-C.

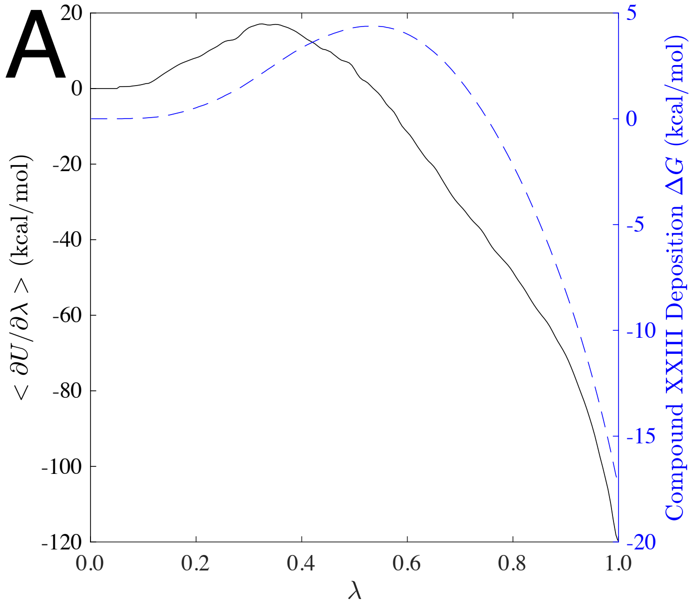

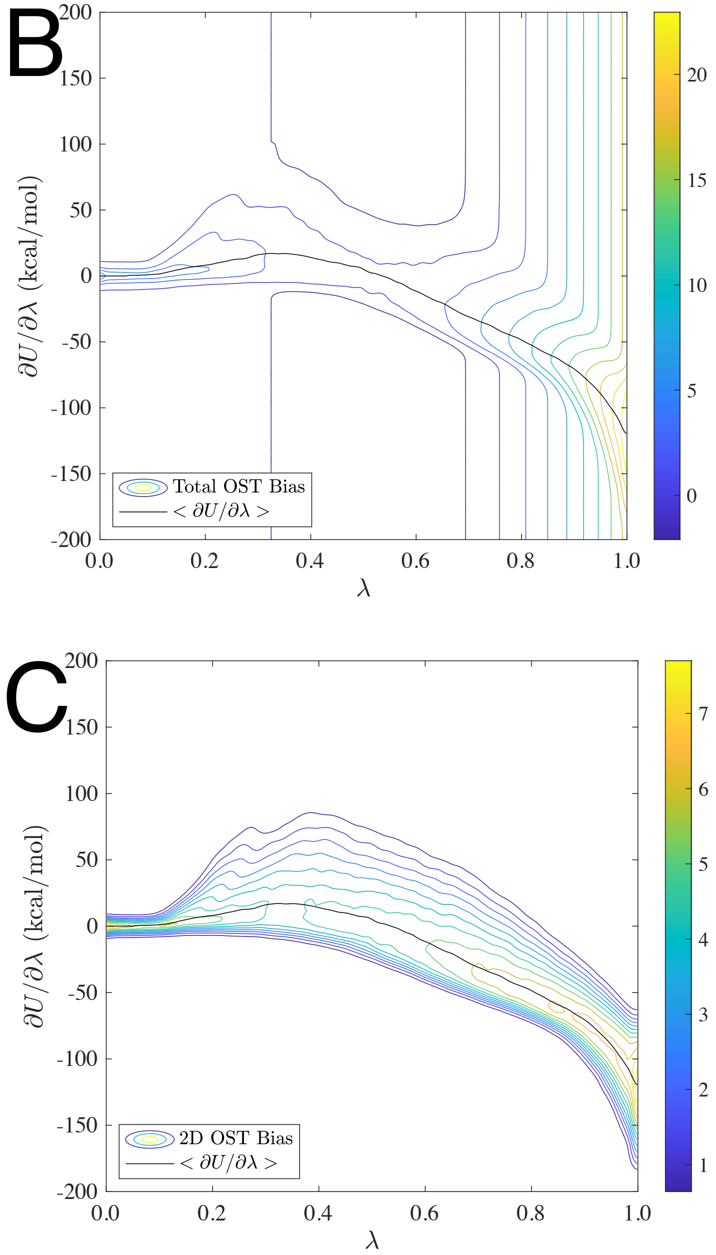


SI Figure 7. Plots illustrating the OST sampling approach for a 10 nsec NVT simulation. Panel A demonstrates the ensemble average partial derivative of the potential energy with respect to $\lambda$ (given by $\langle \partial U/\partial \lambda \rangle$) and overall deposition free energy difference. Note that the $\langle \partial U/\partial \lambda \rangle$ curve is smoother than for the 1 nsec NPT simulation used to generate Figure 6A in the main text. Panel B is a contour plot of the total orthogonal space tempering bias along with the value of $\langle \partial U/\partial \lambda \rangle$ as a function of $\lambda$ for the 10 nsec alchemical NVT deposition/sublimation simulation. Panel C contains a contour plot of only the 2D orthogonal space tempering bias along with the value of $\langle \partial U/\partial \lambda \rangle$ as a function of $\lambda$ for the same simulation.
