# Supplementary material for: Crystal Polymorph Search in the NPT Ensemble via a Deposition/Sublimation Alchemical Path
Source: Cryst Growth Des. 2024 Mar 9;24(8):3205–17. doi: 10.1021/acs.cgd.3c01358 (PMC11036363; doi:10.1021/acs.cgd.3c01358)
Supplement: Supplementary file 2 — cg3c01358_si_002.zip [file cg3c01358_si_002.zip › ACS_SI/Supplemental-Information.pdf]

Supplemental Information for:

Crystal Polymorph Search in the NPT Ensemble via a Deposition/Sublimation Alchemical Path

Aaron Nessler (ORCID: 0009-0004-2502-6204)[1], Okimasa Okada[2], Yuya Kinoshita[3], Koki Nishimura[3], Hiroomi Nagata[4], Kaori Fukuzawa (ORCID: 0000-0001-5357-8250) [5], Etsuo Yonemochi (ORCID: 0000-0001-5255-5129)[6], and Michael J. Schnieders (ORCID: 0000-0003-1260-4592)[1,7,*]

[1]University of Iowa Department of Biomedical Engineering, 103 South Capitol Street, 5601 Seamans Center for the Engineering Arts and Sciences, Iowa City, IA, 52242

[2]Sohyaku. Innovative Research Division, Mitsubishi Tanabe Pharma Corporation, Japan

[3]Analytical Development, Pharmaceutical Sciences, Takeda Pharmaceutical Company Limited, 2-26-1, Muraoka-Higashi, Fujisawa, Kanagawa 251-8555, Japan

[4]CMC Modality Technology Laboratories, Production Technology and Supply Chain Management Division, Mitsubishi Tanabe Pharma Corporation, Japan

[5]Graduate School of Pharmaceutical Sciences, Osaka University, 1-6 Yamadaoka, Suita, Osaka 565-0871, Japan

[6]Department of Physical Chemistry, School of Pharmacy and Pharmaceutical Sciences, Hoshi University, 2-4-41 Ebara, Shinagawa-ku, Tokyo 142-8501, Japan

[7]University of Iowa Department of Biochemistry, 51 Newton Road, 4-403 Bowen Science Building, Iowa City, IA, 52242

*Corresponding Author: michael-schnieders@uiowa.edu

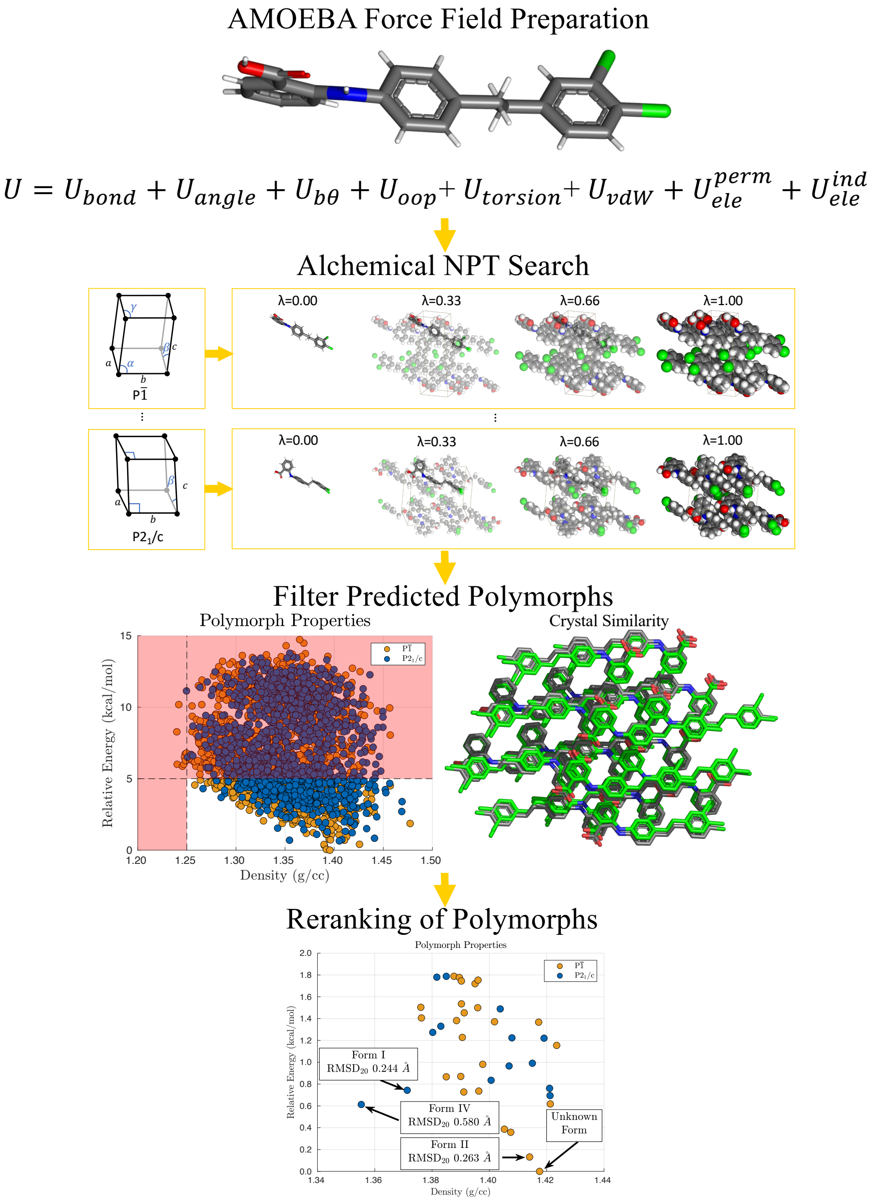

SI Figure 1. A diagram describing the steps used to predict the crystal polymorphs. After parameterization, the polymorph search method generates space groups for the input structure. After the thermodynamics simulation in the NPT ensemble, each snapshot is minimized and then filtered (*i.e.*, using potential energy and density). Similar structures are then removed using the PAC algorithm. Finally, the polymorphs can be reranked with a more accurate method such as DFT.

SI Table 1. Predicted polymorphs with $RSMD_{20}$ to experiment less than 1.5 Å were followed through the pipeline. Entries with N/A were filtered out either due to energy/density cutoffs or PAC similarity clustering. The entry highlighted in blue does not match an experimental polymorph but given its energy and density it is potentially a novel polymorph that has yet to be discovered.

Space Group	Run	Job	Structure	$RMSD_{20}$ PAC (Å)	Relative Energy (kcal/mol)	Density QM (g/cm^3)	Relative Energy (kcal/mol)	Density QM (g/cm^3)	$RMSD_{20}$ QM (Å)
$P\bar{1}$	2	93	137	1.13	0.894	1.46	N/A	N/A	N/A
	2	55	36	1.15	0.078	1.48	N/A	N/A	N/A
	3	49	80	1.19	-0.780	1.49	0.13	1.41	0.27
	3	49	79	1.16	N/A	N/A	N/A	N/A	N/A
	3	10	131	1.15	-0.222	1.49	N/A	N/A	N/A
	4	82	58	1.12	-0.515	1.50	N/A	N/A	N/A
	5	32	106	1.12	N/A	N/A	N/A	N/A	N/A
	1	09	193	8.58	0.000	1.45	0.00	1.42	8.12

Space Group	Run	Job	Structure	$RMSD_{20}$ PAC (Å)	Relative Energy (kcal/mol)	Density QM (g/cm^3)	Relative Energy (kcal/mol)	Density QM (g/cm^3)	$RMSD_{20}$ QM (Å)	Form
$P2_1/c$	2	81	82	1.25	0.00	1.45	0.00	1.37	0.66	A
	2	81	83	1.24	N/A	N/A	N/A	N/A	N/A	A
	5	79	94	0.78	1.12	1.38	N/A	N/A	N/A	D
	5	79	98	0.57	0.02	1.41	-0.74	1.35	0.58	D
	5	79	99	0.60	0.54	1.39	N/A	N/A	N/A	D
	5	34	45	1.17	0.06	1.43	N/A	N/A	N/A	A
	5	34	49	1.17	N/A	N/A	N/A	N/A	N/A	A
	5	34	50	1.17	N/A	N/A	N/A	N/A	N/A	A

Impact of Orthogonal Space Tempering

Two sets simulations were performed using the NVT ensemble to access the use of OST. Each contained ten trials of 100 simulations sampling for 10 nsec. Both sets were supplied the experimental lattice parameters to simplify the search procedure to atomic coordinates within a fixed lattice. A random translation and rotation were applied to the molecule in vacuum prior to the alchemical NPT simulation beginning. On average, 0.4% of the simulations without the 2D OST bias reached the experimental structure (*i.e.*, within an $RMSD_{20}$ of 1.5 Å) whereas 0.8% were successful when the 2D OST bias was included used. These results suggest that the 2D OST bias offers a ~2x improvement in efficiency for Compound XXIII when the search is limited to atomic coordinates. Representative results from using OST with the 2D bias are given in SI Figures 2 through 4 below.

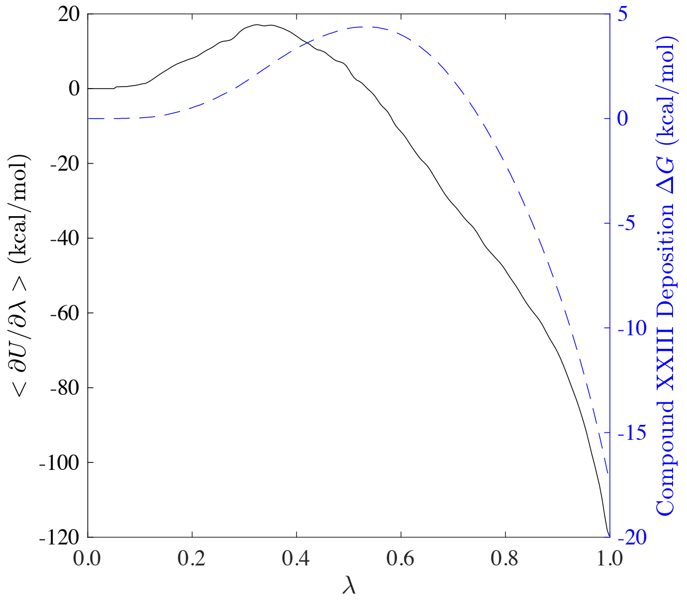

SI Figure 2. The ensemble average partial derivative of the potential energy with respect to λ (given by $\langle \partial U/\partial \lambda \rangle$) and overall deposition free energy difference from a 10 nsec NVT simulation. Note that the $\langle \partial U/\partial \lambda \rangle$ curve is smoother than for the 1 nsec NPT simulation used to generate Figure 7A.

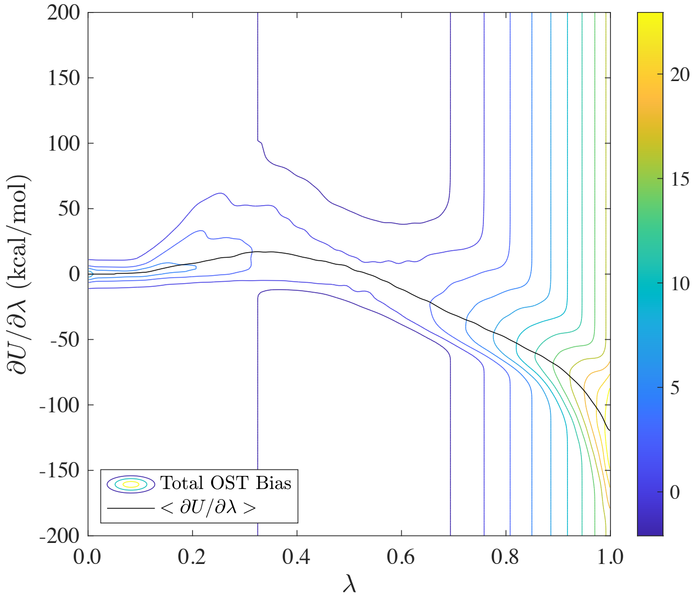

SI Figure 3. A contour plot of the total orthogonal space tempering bias along with the value of $\langle \partial U/\partial\lambda \rangle$ as a function of λ for a 10 nsec alchemical NVT deposition/sublimation simulation.

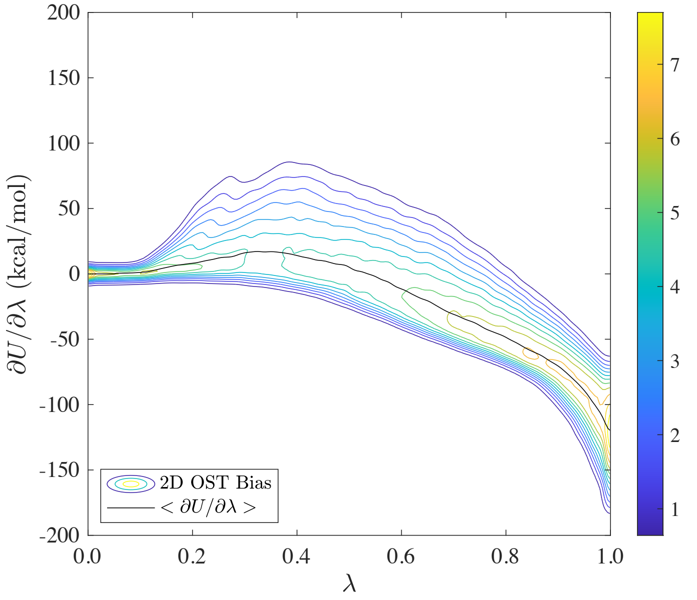

SI Figure 4. A contour plot of the only the 2D orthogonal space tempering bias and along with the value of $\langle \partial U/\partial\lambda \rangle$ as a function of λ for a 10 nsec alchemical NVT deposition/sublimation simulation.
